# Ambient temperature CO_2_ fixation to pyruvate and subsequently to citramalate over iron and nickel nanoparticles

**DOI:** 10.1038/s41467-023-36088-w

**Published:** 2023-02-02

**Authors:** Tuğçe Beyazay, Kendra S. Belthle, Christophe Farès, Martina Preiner, Joseph Moran, William F. Martin, Harun Tüysüz

**Affiliations:** 1 Max-Planck-Institut für Kohlenforschung, Mülheim an der Ruhr, Germany; 2 Faculty of Geosciences, Utrecht University, Department of Ocean Systems, Royal Netherlands Institute for Sea Research (NIOZ), Yerseke, The Netherlands; 3 Université de Strasbourg, CNRS, ISIS UMR 7006, Strasbourg, France; 4 Institute of Molecular Evolution, University of Düsseldorf, Düsseldorf, Germany

**Keywords:** Origin of life, Nanoparticles, Heterogeneous catalysis

## Abstract

The chemical reactions that formed the building blocks of life at origins required catalysts, whereby the nature of those catalysts influenced the type of products that accumulated. Recent investigations have shown that at 100 °C awaruite, a Ni_3_Fe alloy that naturally occurs in serpentinizing systems, is an efficient catalyst for CO_2_ conversion to formate, acetate, and pyruvate. These products are identical with the intermediates and products of the acetyl-CoA pathway, the most ancient CO_2_ fixation pathway and the backbone of carbon metabolism in H_2_-dependent autotrophic microbes. Here, we show that Ni_3_Fe nanoparticles prepared via the hard-templating method catalyze the conversion of H_2_ and CO_2_ to formate, acetate and pyruvate at 25 °C under 25 bar. Furthermore, the ^13^C-labeled pyruvate can be further converted to acetate, parapyruvate, and citramalate over Ni, Fe, and Ni_3_Fe nanoparticles at room temperature within one hour. These findings strongly suggest that awaruite can catalyze both the formation of citramalate, the C5 product of pyruvate condensation with acetyl-CoA in microbial carbon metabolism, from pyruvate and the formation of pyruvate from CO_2_ at very moderate reaction conditions without organic catalysts. These results align well with theories for an autotrophic origin of microbial metabolism under hydrothermal vent conditions.

## Abstract (web summary)

Synthetic Ni-Fe nanoparticles have been demonstrated as catalysts for CO2 fixation to formate, acetate, and pyruvate at higher temperatures. Here, the authors show these can convert pyruvate to citramalate at the ambient temperature, in line with origin of microbial metabolism under hydrothermal vent conditions.

## Introduction

Since the discovery of the Lost City hydrothermal field only 20 years ago^1^, off-ridge vents fed by serpentinizing hydrothermal systems have stood out in the context of life’s origin because of their relatively low temperature, their H_2_-richness, their chemically reactive environments and the nature of inorganic catalysts that naturally occur in such systems^2,3^. The far from equilibrium chemistry of Lost City-type vents results from rock–water interactions that constitute the process of serpentinization. During serpentinization, H_2_ is generated via the reduction of water by ferrous ions present in mineral olivine of ultramafic rocks^4,5^. In H_2_-rich hydrothermal vents, oxides of Fe and Ni (in the form of Fe^2+^ and Ni^2+^) in the crust can also be reduced to their native metal forms^6–8^ or their alloys such as Ni_3_Fe (awaruite)^6,8^, one of the most commonly reported Ni–Fe alloys found in hydrothermal vents^9–12^. The H_2_-rich environment of serpentinizing systems most likely played a critical role in early metabolic evolution^1,13^ because its carbon-fixation potential/ability closely resembles that of the acetyl Coenzyme A (acetyl-CoA) pathway, an ancient, exergonic, linear and H_2_-dependent CO_2_ fixation pathway, the enzymes of which are replete with catalytic Fe and Ni atoms at their active sites^14–16^.

Though CO_2_ reduction in the acetyl-CoA pathway involves simple intermediates and products—formate, acetate, and pyruvate^17,18^—, its catalytic mechanism is complex since CO_2_ is a highly stable molecule. Its reduction to intermediates of the acetyl-CoA pathway requires multiple electron transfers^19–23^ but the equilibrium lies on the side of the organic products under our reaction conditions^24,25^. Under physiological conditions, even the H_2_-dependent reaction to the level of the energy-rich thioester is exergonic with Δ*G*_o_’ = −59 kJ mol^–2^ ^26^. The enzymatic mechanisms of H_2_-dependent fixation of CO_2_ to acetyl-CoA and pyruvate have been studied in detail^24,27–29^ and X-ray structures reveal that the central enzymes of the pathway, carbon monoxide dehydrogenase (CODH) and acetyl-CoA synthase (ACS), harbor transition metal (Ni, Fe) clusters at their active sites^30–32^. The enzymatically catalyzed reduction of CO_2_ to acetyl-CoA intermediates^33–35^ has been studied in considerable detail. Energetic obstacles in the pathway occur at the conversion of CO_2_ to a pterin bound N-formyl group^36^, and the generation of reduced ferredoxin with electrons from H_2_, which requires electron bifurcation^37^. At 1 bar of H_2_, the H_2_-dependent conversion of CO_2_ to formate is at equilibrium^28^.

Although about 10 proteins and an equal number of organic cofactors are required to convert CO_2_ and H_2_ to formate, acetate and pyruvate in the enzymatically catalyzed acetyl-CoA pathway^15,24^, the same products can be obtained at 100 °C without proteins or cofactors, using only Ni_3_Fe as the catalyst^25^. Recent studies have shed light on transition metal catalyzed CO_2_ reduction under simulated hydrothermal vent conditions^25,38–42^. Non-enzymatic CO_2_ fixation to formate, acetate, and pyruvate has been reported using reducing agents including an external electrical source^43–45^, native metals as reductants^40,42^ or molecular hydrogen^25,46^. Furthermore, formation of pyruvate from formate at 250 °C and 1000 bar under CO atmosphere was reported in an iron and sulfur-rich environment^47^. However, high temperatures used in some of these studies might have precluded the accumulation of some biologically relevant products. The physiological significance of the reductive acetyl-CoA pathway for metabolic origins stems from its central role in metabolism^48–51^, where it links CO_2_ fixation with the tricarboxylic acid (TCA) cycle to provide essential intermediates for biosynthesis^52^. However, there are a number of microorganisms that can still grow in the absence of the essential TCA enzyme isocitrate lyase^53^. One of the alternative pathways for these microorganisms to synthesize TCA intermediates is the citramalic acid cycle^54^. This has been well-characterized in the purple bacterium *Rhodosprillum rubrum*, which can grow on acetate via the citramalate (CMA) pathway when CO_2_ is present in the environment^55–57^. Citramalate was also reported as a decomposition product of citric acid under hydrothermal conditions by aqua-thermolytic degradation^58,59^ and CMA pathway has been described in several types of bacteria for the synthesis of branched-chain esters and amino acids^60,61^. Although there have been numerous reports on enzymatic condensation of acetyl-CoA and pyruvate to citramalate in algae^62^, yeasts^63^, and bacteria^64–69^, with citramalyl-CoA occurring in the 3-hydroxypropionate pathway of CO_2_ fixation^24^, the full spectrum of connections between CO_2_ and the citramalic acid cycle is still unresolved.

Here, we show the abiotic synthesis of acetyl-CoA pathway products formate, acetate and pyruvate through CO_2_ fixation at ambient temperature over synthetic Ni-Fe nanoparticles. The ^13^C-labeled pyruvate is further converted to acetate, parapyruvate, and citramalate over Ni, Fe, and Ni_3_Fe nanoparticles. Native Ni, Fe and the hydrothermal alloy awaruite can replace the function of several enzymes in ancient pathways.

## Results and discussion

**Fig. 1:**
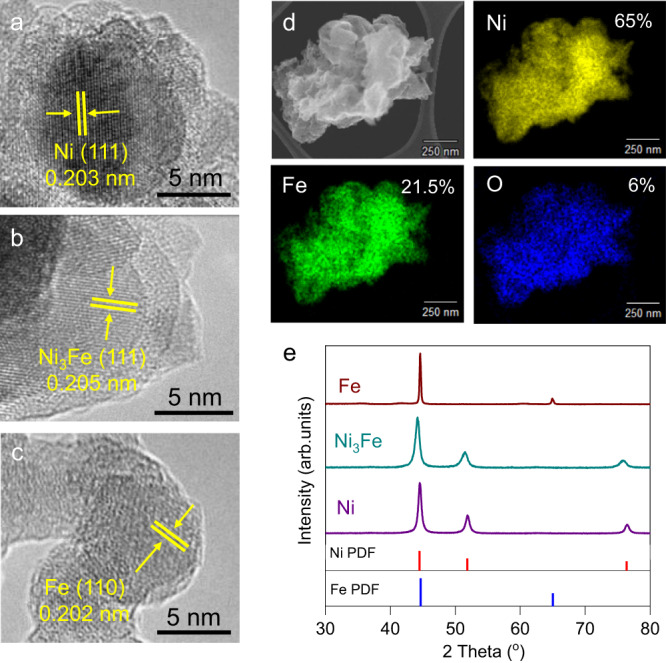
Structural characterization of Ni, Ni_3_Fe, and Fe nanoparticles. HR-TEM images of Ni **a**, Ni_3_Fe **b**, Fe **c** nanoparticles, STEM-EDX mapping of Ni_3_Fe **d**. XRD patterns of Ni, Ni_3_Fe, and Fe **e** with the characteristic reflections of Ni (PDF: 004-0850), Fe (PDF: 006-0696). XRD diagrams and TEM micrographs confirm the formation of reduced Ni-Fe nanoparticles. STEM-EDX mapping of Ni_3_Fe shows that the ratio of Ni to Fe is as desired during the synthesis.

To explore the possible stepwise synthesis of carbon backbones longer than pyruvate from CO_2_ and H_2_, we synthesized nanoparticular forms of Ni, Fe, and their most common hydrothermal vent alloy Ni_3_Fe (awaruite) as metal catalysts to investigate hydrogen-dependent CO_2_ fixation. Ni-Fe nanoparticles were prepared via hard-templating by using spent tea leaves (STL) as a hard template followed by reduction under a hydrogen atmosphere as previously reported^70,71^. Transmission electron microscopy (TEM) surveys confirmed the formation of crystalline nanoparticles in size of 20–30 nm as seen in Supplementary Fig. 1a–c. X–ray Diffraction (XRD) results showed the formation of highly crystalline metallic Ni, Fe, and Ni–Fe phases (Fig. 1e). Since Ni and Fe have similar lattice parameters and X-Ray patterns, we further checked characteristic crystal fringes to confirm the formation of Ni_3_Fe alloy.

High-resolution TEM (HR-TEM) clearly shows lattice fringes of nanoparticles with a spacing of 0.203 nm, 0.205 nm, and 0.202 nm corresponding to Ni (111), Ni_3_Fe (111), and Fe (110), respectively (Fig. 1a–c). For Ni_3_Fe, high-resolution scanning electron microscopy (HR-SEM) micrograph and energy dispersive X-ray (EDX) mapping were also performed to display the uniform distribution of Ni and Fe particles (Fig. 1d). The homogeneous distribution of Ni and Fe with atomic ratio of 3 to 1 confirms the successful synthesis of Ni_3_Fe alloy. N_2_ sorption analyses indicate that the prepared materials show some degree of mesoporosity and their Brunauer–Emmett–Teller (BET) surface areas are found to be around 30 m^2^/g for all samples (Supplementary Fig. 1d). Supplementary Fig. 2 shows thermogravimetric analysis (TGA) of Ni_3_Fe catalyst in order to demonstrate that the carbon-based template was successfully removed from the metal catalyst. TGA was recorded until 900 °C under an air atmosphere and mass spectroscopy was integrated to the instrument to monitor released gas during the heating process. No CO_2_ (m/z: 44) could be detected that could be associated with the combustion of carbon residues. Thus, the templated materials can be implemented as carbon-free catalysts for CO_2_ fixation. Possible metal contaminants associated with the carbon-based template were also investigated by SEM-EDX (Supplementary Fig. 3). Besides Fe and Ni, small quantity of other elements like Ca, Mg, and P were detected (Supplementary Table 1), which are expected to not show any noticeable catalytic activity.

In a previous study, we found that Ni_3_Fe can convert CO_2_ to formate, acetate, and pyruvate at 60–100 °C under a pressure of 25 bar^25^. Considering the relatively low temperature of Archean seawater^72^, we explored in the present study H_2_-dependent CO_2_ fixation at milder conditions by choosing a temperature of 25 °C and pressure of 25 bar (CO_2_ + H_2_ with a ratio of 3:2) at pH 6 (before the addition of CO_2_) in a autoclave reactor (Supplementary Fig. 4). These mild reaction conditions are more in line with biological CO_2_ fixation which typically takes place at ambient temperatures^21^. Concentrations of formed products were determined with high-performance liquid chromatography (HPLC) with a calibration curve using pure standard solutions. The HPLC chromatogram is presented in Supplementary Fig. 5, and the corresponding retention times of the expected compounds are provided in Supplementary Table 2. Based on HPLC analyses, formate, acetate, and pyruvate were obtained with concentrations of 26.72, 0.04, and 0.02 mM, respectively (Supplementary Figs. 6a and 7a). Existence of formate, acetate, and pyruvate were also confirmed by ^1^H-NMR (Supplementary Fig. 8).

Next, we investigated the ability of Ni_3_Fe to reduce CO_2_ in H_2_O (at 25 bar and 25 °C) without the addition of molecular H_2_. After 24 h of reaction time, only formate was obtained at 2.27 mM concentration (Supplementary Figs. 6b and 7b), a ten-fold reduction compared to the same reaction in the presence of H_2_. The formation of formate was further supported by ^1^H-NMR analysis (Supplementary Fig. 9). The outcome supports that the Ni_3_Fe catalyst can act as a reductant for CO_2_ reduction, which goes in line with a recent study that demonstrated direct reducing effect of FeS_x_ hydrothermal minerals^73^. Without the molecular H_2_, neither acetate nor pyruvate was observed at 25 °C. It has been proposed that CO_2_ fixation generates acetate and pyruvate on the metal surface starting from the formyl group^40^. If so, additional H_2_ in the reactor might facilitate further conversion of formate to acetate and pyruvate. A series of control experiments were carried out without metal catalyst or CO_2_ to examine the possible catalytic effect of the reactor or of other impurities. Only trace amounts of formate and acetate were detected by HPLC and ^1^H-NMR in control experiments, with much lower concentrations than in the reactions with CO_2_ and metal catalysts. Pyruvate was not detected in any control experiment (Supplementary Fig. 10). The post-reaction XRD analysis did not show any substantial changes in the bulk structure of the catalyst (Supplementary Fig. 11).

**Fig. 2:**
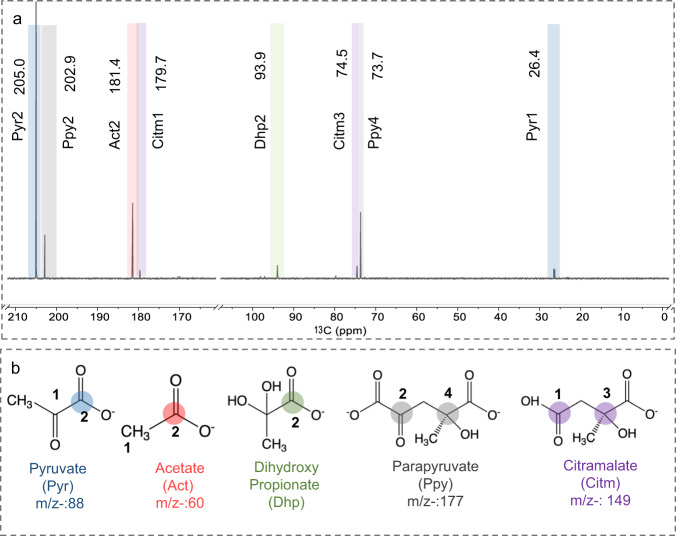
^13^C-labeled products after pyruvate conversion over Ni_3_Fe. ^13^C-NMR spectrum of pyruvate conversion over Ni_3_Fe catalyst after 1 h at 25 °C and peak positions are labeled **a**. Obtained products, pyruvate, acetate, parapyruvate and citramalate, ^13^C-labeled carbons are highlighted **b**. According to ^13^C-NMR, labeled acetate, parapyruvate, and citramalate were obtained after 1 h at 25 °C.

Among the observed H_2_-dependent CO_2_ fixation products, pyruvate is an attractive intermediate in the context of origins of life due to its central role in many anabolic/catabolic pathways^16,74–77^. Therefore, we further studied the ability of a Ni_3_Fe solid catalyst—the same catalyst used for CO_2_ reduction—to convert pyruvate to additional biologically significant products. We used 2-^13^C-labeled pyruvate as a probe molecule since site-specific ^13^C-labeling provides an improvement in both product ^13^C-based NMR analysis and the prediction of reaction mechanism^78^. Initially, the effect of reaction time on product spectrum and distribution over Ni_3_Fe catalyst was studied. Pyruvate (11.35 mM, 1.0 mg/ml) was used as a starting reactant and reactions were carried out at pH 5 over Ni_3_Fe catalyst (0.25 M; total metal concentration is 1 M, 174 mg Ni_3_Fe in 3 mL H_2_O). Reactions were conducted under an aerobic atmosphere by degassing the reaction mixture with argon bubbles for 5 min to eliminate possible carbon contamination from air. A short reaction time of 1 h under ambient conditions resulted in the formation of ^13^C-acetate (IUPAC position C1), ^13^C_2_-parapyruvate (positions 2 and 4), and ^13^C_2_-citramalate (position 1 and 3), which were detected in ^13^C-NMR spectra (Fig. 2a). Electron Spray Ionization Mass Spectroscopy (ESI-MS) further confirmed the isotope-related increase in the mass of ^13^C-labeled products in negative ionization mode. For carboxylic acids, their deprotonated molecular ions [M-H]^-^ gave the highest intensity in negative mode^79^. As ESI-MS is a soft ionization method, it induces little molecular fragmentation^80^ and can therefore deliver insights about possible metal-ligand complexes^81^. It was seen that the mass increased by 2 amu (m/z-149.037), 2 amu (m/z-177.032), and 1 amu (m/z-60) for citramalate, parapyruvate and acetate, respectively. (Supplementary Fig. 12a). We could not detect any non-^13^C labeled citramalate (m/z- 147) or acetate (m/z- 59) after 1 h of the reaction time based on ESI-MS results. We did not observe the formation of any targeted product in the absence of the metal catalyst (Supplementary Fig. 12b). This clearly indicates that ^13^C-pyruvate was the only carbon source for the formation of acetate and citramalate in these reactions.

**Fig. 3:**
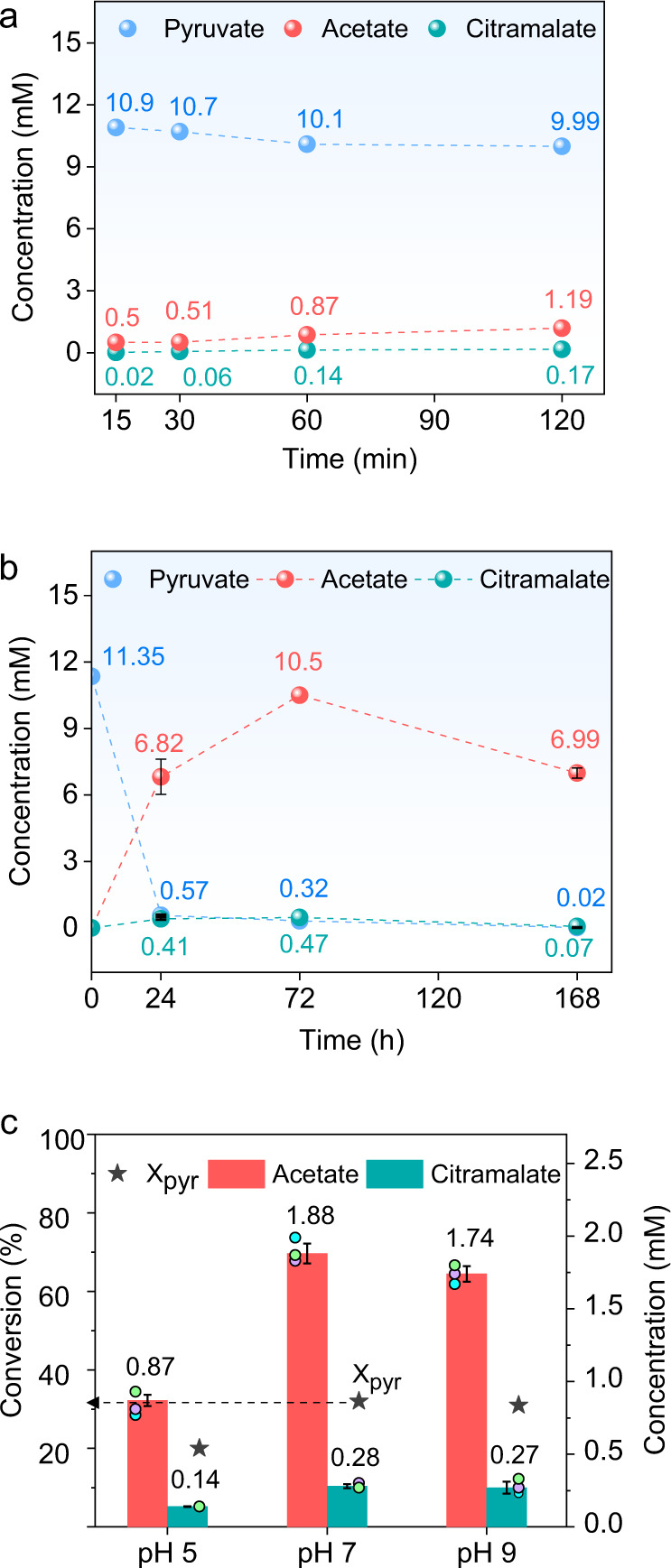
Pyruvate conversion performance of Ni_3_Fe catalyst. Product concentrations from pyruvate conversion (11.35 Mm initial concentration) over Ni_3_Fe catalyst at 25 °C during different reaction times of 15–120 min **a**, and 24–168 h **b**. Pyruvate conversion and product concentrations with different starting pH values after 1 h at 25 °C over Ni_3_Fe catalyst **c**. Pyruvate conversions are represented as X_pyr_ in the figure. For **a**–**c**, initial concentration of pyruvate is always 11.35 mM (1.0 mg/ml) and initial concentrations of acetate and citramalate are zero. Data in **b**, **c** are presented as mean values. Error bars correspond to the standard deviation of three independent reactions.

Concentrations of obtained products were determined by HPLC technique, as presented in Fig. 3. The initial concentration of pyruvate was 11.35 mM (1.0 mg/ml) and 11% of the pyruvate was consumed after 1 h of the abiotic catalytic reaction by forming 0.87 mM of acetate and 0.14 mM of citramalate (Fig. 3a). The presence of ^13^C in acetate after the catalytic reaction shows the direct formation of acetate from pyruvate via oxidative decarboxylation in the presence of H_2_O over the metal catalyst. The products of a parallel experiment, carried out under similar conditions in an autoclave under 2 bar of Ar at 20 °C, were investigated by NMR to check for C–C bond cleavage (Supplementary Fig. 13). After 1 h of catalytic reaction, the autoclave was directly connected to a gas chromatograph with the released gaseous products directly analyzed, whereby 5 ppm of CO_2_ was detected. This supports oxidative decarboxylation of pyruvate. Non-enzymatic pyruvate conversion to acetate has been reported at 70 °C^82^ and with the addition of NAD^+^^83^. Our present findings show that the C–C bond cleavage can occur also at 25 °C under ambient pressure over a Ni–Fe catalyst that naturally occurs in serpentinizing hydrothermal vents.

In order to gain some more insights about the product spectrum and intermediates for conversion of pyruvate to citramalate, the effect of the reaction time on the product distribution was studied further. First, the reaction was carried out for 15 min, 30 min, 1 h and 2 h and the results are presented in Fig. 3a. After a very short reaction time of 15 min, 0.02 mM citramalate could be detected by HPLC. Increasing the reaction time to 30 min and 2 h resulted in increased amounts of citramalate and acetate. After a reaction time of 15 or 30 min, the only detected soluble products were acetate, parapyruvate, and citramalate according to ^13^C-NMR (Supplementary Fig. 14). An increase in peak intensities for parapyruvate and citramalate was observed in ^13^C-NMR spectra. No additional soluble side or intermediate products were detected based on systematic ^13^C-NMR, ESI-MS, and HPLC analyses.

After further increase of reaction time to 24 h, 94.8% of pyruvate was consumed and the amount of citramalate and acetate was increased to 0.41 mM and 6.82 mM, respectively (Fig. 3b). Existence of citramalate, acetate, and pyruvate was further confirmed by key long-range correlations in 2D NMR Heteronuclear Multiple Bond Correlation Spectroscopy (HMBC) (Supplementary Fig. 15). Parapyruvate was also observed with ^13^C-NMR with lower relative intensity than citramalate after 24 h (Supplementary Fig. 16, shown in the zoom area), it was not detected with ESI-MS after 24 h due to its low concentration (Supplementary Fig. 17). When the reaction time was increased to 72 h, the citramalate amount stayed roughly constant while parapyruvate completely disappeared (Supplementary Figs. 16b and 17). The initial pyruvate was almost completely consumed after 168 h of the reaction time (0.02 mM). On the other hand, the amount of citramalate and acetate was also decreased to 0.07 mM and 6.83 mM, respectively. This indicates that the obtained products might be further converted to other products like CO_2_. As stated above, CO_2_ was detected in the gas phase even after the reaction time of only 1 h according to GC analysis. Because the reaction takes place in water, we monitored the aqueous CO_2_ change over the reaction time by HPLC. For this purpose, in a control experiment, deionized water was saturated with CO_2_, which gave a characteristic peak at 5.5 min and two strong negative peaks at 17.5 and 18.5 min (Supplementary Fig. 18a). After 168 h of reaction time of pyruvate over Ni_3_Fe catalysts, we could clearly observe formation of dissolved CO_2_ by HPLC (Supplementary Fig. 18b).

Another crucial parameter in prebiotic chemistry is the pH of the reaction environment. As mentioned above, the formation of citramalate might be the consequence of either a condensation reaction between the produced acetate and pyruvate or of a decarboxylation of the produced parapyruvate. Parapyruvate is a well-known and widely reported homo-aldol condensation product of pyruvate^84–86^, which is more favored under alkaline conditions^87^. To investigate the role of pH for pyruvate conversion to citramalate, two more parallel reactions were performed at pH 7 and 9 with the addition of KOH, and the outcomes are presented in Fig. 3c. An increase of pH from 5 to 7 augmented the conversion and amount of products considerably, with 1.88 mM acetate and 0.28 mM citramalate being obtained after 1 h over Ni_3_Fe. Further increase of pH to 9 did not significantly affect the conversion and concentrations of citramalate and acetate. However, ^13^C-NMR and ESI-MS spectra of the reaction at pH 9 displayed a slight increase in the peak ratio of parapyruvate compared to citramalate (Supplementary Figs. 19 and 20). That the concentration of citramalate remained constant under alkaline conditions (determined by HPLC) might be attributable to the higher stability of parapyruvate, the possible intermediate in citramalate formation. Since the citramalate amount remained stable at pH 9, a control experiment without any catalyst at pH 9 indicated no pyruvate conversion, demonstrating an essential role for the solid catalyst even under an alkaline environment (Supplementary Figs. 19 and 20).

Inductively coupled plasma—optical emission spectrometry (ICP-OES) was used to analyze possible leached metal ions during the reactions of pyruvate to provide some insights about the catalytic influence of the leached metal species. ICP-OES results indicated that 0.183 µg/ml Fe (0.549 µg in 3 mL) and 0.044 µg/ml (0.132 µg in 3 mL) Ni were leached into the solution with 1.0 mg/ml pyruvate after 1 h (Supplementary Table 3). Higher amount of leached Fe compared to Ni from Ni_3_Fe catalyst is not surprising, considering the standard oxidation potential of E^0^ of Fe/Fe^2+^ is +440 mV *vs* NHE and E^0^ of Ni/ Ni^2+^ is +230 mV *vs* NHE. The initial amount of the catalyst was 174 mg in 3 mL and 0.681 µg metal in total was leached after 1 h into the reaction solution. Although the amount of leached metal ions is not very high, another reaction was performed to investigate the catalytic effect of leached Ni-Fe species in the solution. After 1 h of reaction, the solid Ni_3_Fe was hot-filtered and the reaction was carried out for 24 h in the absence of any solid catalyst. As seen in Supplementary Fig. 21, HPLC revealed a negligible effect of the leached Ni–Fe species on pyruvate conversion, indicating that pyruvate conversion is taking place on the surface of solid Ni_3_Fe catalyst.

To investigate possible reaction pathways and correlation between produced acetate and pyruvate in more details, we conducted further experiments by using both ^12^C-acetate and ^13^C-pyruvate as starting substrates under the same catalytic reaction conditions in the presence of Ni_3_Fe catalyst. After 1 h of shaking with Ni_3_Fe catalyst, only 1, 3-^13^C_2_-citramalate was detected with both ^13^C-NMR and ESI-MS (m/z-: 149) (Supplementary Figs. 22 and 23). The lack of detectable m/z-: 148 indicates that citramalate stems from only parapyruvate rather than the condensation of acetate and pyruvate (Supplementary Fig. 23). ^13^C-NMR results confirm the existence of both ^12^C-acetate and ^13^C-acetate after 1 h of the catalytic reaction. According to the HPLC analysis result, the pyruvate concentration decreased from 5.67 mM to 0.79 mM after 1 h of reaction time. Conversely, the amount of acetate increased from 4.16 mM to 6.32 mM. This result indicates that 2-^13^C-pyruvate is the only carbon source of citramalate and co-utilization of pyruvate and acetate did not lead to more citramalate production (Supplementary Fig. 24a). Further control experiments were also carried out in the absence of metal catalysts under the same reaction conditions. ^13^C-pyruvate, ^12^C-acetate, and ^13^C-2,2-dihydroxy-propanoate were detected without metal catalyst after 1 h and obtained concentrations of pyruvate and acetate did not change after 1 h of shaking (Supplementary Fig. 24b). Importantly, no C-C bond cleavage or formation of products was detected without a metal catalyst. After observing that acetate did not contribute to citramalate production, the stability of acetate alone was also investigated under these reaction conditions over the Ni_3_Fe catalyst. The amount of acetate remained stable after 1 h of shaking with or without the metal catalyst (Supplementary Fig. 25).

**Fig. 4:**
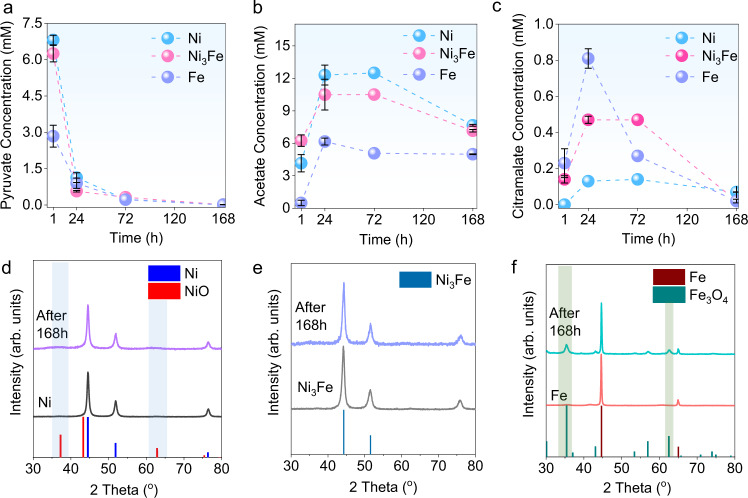
Pyruvate conversion performances of Ni-Fe catalysts and their post-reaction characterizations. Concentrations of pyruvate **a**, acetate **b**, and citramalate **c** from pyruvate conversions (initial pyruvate concentration is 11.35 mM) over Ni, Ni_3_Fe, and Fe catalysts during different reaction times at 25 °C. Initial concentrations of citramalate and acetate are zero. Both citramalate and acetate concentrations were increased until 24 h over all metal catalysts. While the concentrations remained the same until 72 h over Ni and Ni_3_Fe catalyst, the significant decrease of obtained products was observed over Fe catalyst after 72 h. Concentration of pyruvate was decreased over time with all metal catalysts until it completely vanished after 168 h. XRD patterns of Ni **d** (PDF Ni: 00-004-0850 and NiO: 00-044-1159), Ni_3_Fe **e**, and Fe **f** (PDF Fe: 00-006-0696 and Fe_3_O_4_: 00-019-0629) before and after the reaction show the change in bulk structures of catalysts. Data in **a**–**c** are presented as mean values of three independent experiments and error bars represent standard deviations.

Afterward confirming that Ni_3_Fe can catalyze citramalate formation from pyruvate, the roles of iron and nickel and their synergistic effects were further investigated. For this purpose, the same experiments were also conducted over native Ni^0^ and Fe^0^ nanoparticles by using 11.35 mM (1.0 mg/ml) of pyruvate as reactant. As seen in Fig. 4a, after 1 h of reaction time by simple shaking the reactants and solid catalyst at room temperature, native Fe showed the highest conversion of pyruvate. The pyruvate concentration decreased significantly in the first 24 h over all metal catalysts. After 168 h, almost all pyruvate was consumed. The highest selectivity towards acetate was over Ni, which reached a maximum after 24 h (Fig. 4b). While the acetate amount remained constant over Ni and Ni_3_Fe catalysts, it decreased over Fe until 72 h. Similar to acetate, the amount of citramalate also reached a maximum value after 24 h (Fig. 4c). No citramalate formation over Ni^0^ after 1 h was detected. While the amounts of acetate and citramalate stayed almost constant for Ni and Ni_3_Fe after 72 h, concentrations decreased drastically over the Fe catalyst (Fig. 4c). The amount of all products significantly decreased and pyruvate was completely consumed over all metal catalysts after 168 h. Additionally, 0.34 mM of ^12^C-formate was obtained over the Fe catalyst after 168 h.

Gas product analyses of the reactions over metal catalysts were further investigated to detect possible decomposition products. Standard CO_2_ gas was measured first with GC and a control reaction was performed with 1.0 mg/ml pyruvate in the absence of a metal catalyst for 1 h (Supplementary Fig. 26). The amount of CO_2_ after 1 h without the addition of a metal catalyst was negligible compared to reactions with metal catalysts. Pyruvate conversion over Fe^0^ after 1 h showed the formation of CO_2_ which significantly increased after 168 h (Supplementary Fig. 27). The CO_2_ value obtained over Fe^0^ after 168 h was much higher than with the Ni^0^ catalyst (Supplementary Fig. 28). Besides CO_2_, the formation of H_2_ was observed over Ni^0^ as a C–H cleavage product. The generated electron from the oxidative decarboxylation of pyruvate could also play a role for H_2_ formation from water splitting, however, electron flux during the pyruvate conversion reaction was not observed with any probe molecules. HPLC analysis after 168 h further revealed higher dissolved CO_2_ amounts over Fe^0^ as compared to Ni_3_Fe and Ni^0^ as catalysts (Supplementary Fig. 29). The reasons behind differing catalytic behaviors and alteration of catalysts will be discussed below in the section on post-reaction catalyst characterization.

To further study the role of nanoparticles, an additional experiment was performed by using commercial bulk Fe as catalyst for pyruvate conversion. The pyruvate amount decreased significantly from 9.1 mM to 0.11 mM and 0.14 mM citramalate was obtained after 1 h of the reaction over Fe nanoparticles. However, the pyruvate amount was not changed significantly over bulk Fe catalyst and no citramalate was detected (Supplementary Fig. 30). This suggests that the small particle size and high surface area of nanoparticles provided large numbers of active centers to convert pyruvate to citramalate.

Post-reaction catalyst characterization sheds light on the catalysts’ alteration as well as active catalytic centers. The XRD analysis indicated a slight oxidation of Ni and Fe metals after 1 h, while further increasing the reaction time barely affects the bulk crystal structure of Ni (Fig. 4d). However, the XRD pattern of the Fe catalyst after 168 h displayed reflections from Fe_3_O_4_ in addition to metallic iron (Fig. 4f). Ni_3_Fe alloy was more resistant to oxidation and kept its initial crystal structure even after 168 h, indicating superior stability of the alloy over the native metals (Fig. 4e) as a catalyst. In order to explore the alteration of surface structure of the Ni_3_Fe catalyst, fresh and spent catalysts after 168 h reactions were investigated by X-Ray photoelectron spectroscopy (XPS). XPS spectrum of Ni 2*p* core-level before the reaction shows characteristic peaks at 855.6 and 852.1 eV, which correspond to Ni(OH)_2_ and metallic Ni^0^, respectively^88^. Disappearance of the peak at 852.1 eV after the catalytic reaction indicates the oxidation of active Ni^0^ centers on the catalyst surface. The XPS spectrum of Fe 2*p* shows a similar trend as Ni 2*p*; the distinctive metallic Fe is detectable around 706 eV^89^ before the reaction, however, it disappeared in 168 h samples (Supplementary Fig. 31). Even though the Ni_3_Fe bulk structure remained similar after 168 h according to XRD results, XPS surface analysis indicated the oxidation of active metal centers after the catalytic reaction. Such surface oxidation is expected in light of the fact that the catalytic reactions have been performed in aqueous pyruvate solution. Native metals like Fe and Ni are known to react with oxygen in water and air moisture to form oxide and hydroxide shells. Further studies need to be conducted to reveal the role of different surface sites for the catalytic conversion of pyruvate to citramalate and its further decomposition to other hydrocarbons and CO_2_.

Since synthesized citramalate vanished after reaction times of 168 h, we further investigated the direct decomposition of citramalate over metal catalysts using pure citramalate as reactant. When the control experiment is conducted without any catalyst, the amount of citramalate did not change as determined by HPLC (Supplementary Fig. 32a). After 1 h, the Ni_3_Fe catalyst converted about 27% of citramalate (Supplementary Fig. 32b) while ca. 82% citramalate was converted over Fe^0^ catalyst (Supplementary Fig. 33b). The dissolved CO_2_ peaks in the HPLC chromatographs verified that citramalate decomposed to CO_2_ even after 1 h. In order to detect possible intermediate products, one additional reaction was performed for 30 min over Fe^0^, which causes the fastest decomposition of citramalate. The HPLC trace taken after 30 min of reaction time revealed the formation of formate and acetate from direct catalytic decomposition of citramalate (Supplementary Fig. 33a). Outcomes from pure citramalate decomposition over native metals are consistent with pyruvate conversion reactions in light of the fact that citramalate produced from pyruvate decreased faster over Fe^0^ than over Ni_3_Fe.

**Fig. 5:**
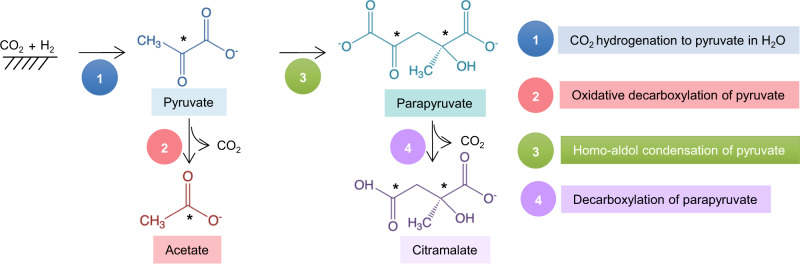
Possible reaction pathway of CO_2_ fixation to citramalate. The product of CO_2_ fixation with H_2_, pyruvate, converts to acetate and releases CO_2._ Parapyruvate is obtained via homo-aldol condensation of pyruvate. Next, parapyruvate converts to citramalate, which yields CO_2_ as a side product. All ^13^C-isotope labeled atoms in the compounds are shown as * in the figure.

The formation of surface-bound C_1_-C_3_ products of CO_2_ fixation over Ni and Fe-based catalysts (including formate, acetate, and pyruvate) has previously been reported^23,25,40^. Here, we reveal that pyruvate can condense to form more complex molecules over solid iron and nickel-based catalysts. Pyruvate formed from CO_2_ can undergo aldol condensation to parapyruvate in a catalyst-dependent manner, the C_6_ product is further transformed to citramalate through C–C bond cleavage and release of CO_2_ at room temperature and atmospheric pressure (Fig. 5). Citramalate seems to be unstable during the catalytic reaction; it is further decomposed to acetate, formate and CO_2_. In modern metabolism, citramalate is a key intermediate in various anaplerotic routes that feed carbon backbones into the TCA cycle^54^, and it can be easily formed over native metals without enzymes. Overall, the solid Ni–Fe catalyst functions not only in a manner similar to the acetyl-CoA pathway^25^ but also similar to citramalate synthase^54^.

We have shown the abiotic synthesis of pyruvate through CO_2_ fixation and its further conversion to citramalate over nickel and iron-based solid catalysts. Hydrothermal vent minerals, Ni, Fe, and Ni_3_Fe, can catalyze both the synthesis of acetyl-CoA pathway products from CO_2_ and further conversion of pyruvate to parapyruvate and citramalate, steps that require many enzymes in the metabolism of modern microorganisms. Investigating the influence of both reaction time and metal catalyst on product distribution and concentration using ^13^C isotope-labeled pyruvate as the substrate showed that reactions between surface-bound C_1_-C_3_ products obtained via H_2_-dependent CO_2_ fixation reactions result in the formation of larger and biologically relevant carbon backbones over the native metal catalyst. Native metals and the hydrothermal alloy awaruite can replace the function of several enzymes in ancient pathways. They are likely older than the enzymatic pathways and may have prepatterned the H_2_-dependent enzymatic pathways that eventually evolved to form the core of chemolithoautotrophic metabolism in modern anaerobes. This points to a natural tendency of the central reactions of ancient carbon metabolism in autotrophs to unfold in the presence of H_2_, CO_2_ and transition metal catalysts that are naturally formed in serpentinizing hydrothermal vents.

## Methods

### Synthesis of Ni–Fe Alloy nanoparticles

Ni(NO_3_)_2_·6H_2_O ( ≥ 97%) and Fe(NO_3_)_3_·9H_2_O ( ≥ 98%) were purchased from Sigma-Aldrich. Spent tea leaves (Goran Mevlana) were utilized as a hard template to obtain metal nanoparticles^70,71^. Firstly, tea leaves were repeatedly washed with distilled water at 80 °C until the washing water color is clear and washed leaves were dried at 80 °C. Dry leaves were immersed into a 0.1 M aqueous solution of Ni (NO_3_)_2_ and Fe (NO_3_)_3_ (60 mL H_2_O). The mass ratio between tea leaves and total metal precursor was set to 2:1 ratio. Ni to Fe ratio in the catalyst was adjusted to 3:1 by tuning the mole ratio between Ni and Fe salt precursors. Solution was mixed for 2 h at room temperature. Upon drying at 80 °C, the carbon-based tea leaves template was calcined at 550 °C for 6 h to obtain metal oxide nanoparticles. Acid washing was performed with shaker IKA KS 130 basic laboratory shaker (IKA® Werke GmbH & Co. KG, Staufen, Germany). After washing with 0.1 M HCl for 2 h (only Fe catalyst was washed for 4 h) to remove impurities (such as Mg, Ca etc.) from the tea template, the final product was obtained after washing the solid with distilled H_2_O for three times. Centrifugation for each washing was performed with Sigma 3–30 KS, (Sigma Laborzentrifugen GmbH, Germany) at 11270 × *g* for 10 min. Synthesized metal oxides were subsequently annealed under 10% H_2_/Ar gas flow with a flow rate of 100 ml/min) at 500 °C for 2 h to obtain reduced metal particles. Finally, the surface passivation process with Ar/Air-gas flow was performed at room temperature to prevent the complete oxidation of metals under air. The H_2_-reduction process was performed just before the reaction to use freshly reduced metal nanoparticles.

### Structural characterization of nanoparticles

Crystal structures of Ni–Fe nanoparticles were analyzed with the X-ray diffraction (XRD) method. Stoe theta/2theta diffractometer in Bragg–Brentano geometry with Cu K Kα1/2 radiation was used to obtain XRD diagrams. Since surface area and porosity of the catalyst are essential parameters to correlate with catalytic performance, Ni–Fe particles were examined with N_2_ sorption with 3Flex Micrometrics at 77 K. Samples were degassed at 150 °C for 10 h before the measurement. Brunauer–Emmett–Teller (BET) surface areas were determined from the relative pressure range between 0.06 and 0.2. Transmission electron microscopy (TEM) images of samples were taken at 100 kV with Hitachi H-7100 electron microscope. High-resolution TEM (HRTEM) and scanning electron microscopy (SEM) images were obtained with HF-2000 and Hitachi S-5500 microscopes, respectively. Thermogravimetric analysis-mass spectroscopy (TG-MS) was performed in order to analyze possible carbon contamination in the catalyst under synthetic air until 900 °C with 10 K/min heating rate. TG-MS result was obtained with Netzsch STA 449 F3 Jupiter connected to QMS 403D (Netzsch) mass spectrometer.

### CO_2_ fixation reactions

Autoclaves made of Mo–Ni alloy were used as pressure reactors. An inlet made of PTFE was used to prevent possible contamination and the catalytic effect coming from autoclaves made of Mo–Ni alloy. For each reaction, a total amount of 1 M metal (1 M Ni, 0.25 M Ni_3_Fe, and 1 M Fe) was used in 3 mL Milli-Q water. To prevent contamination, we did not use any organic buffers in our experiments. Prior to experiments, the pressure reactor was purged 3 times with Ar (5 bar) to prevent cross-contamination from the air and distilled water. Later, the autoclave was pressurized to 25 bar with a CO_2_ + H_2_ mixture (3:2 ratio) as optimized gas mixture based on our previous study^35^. Reactions were performed at 25 °C for 24 h. The reaction without the addition of molecular H_2_ was performed with 25 bar of CO_2_ at 25 °C with 1 M Ni_3_Fe in H_2_O. For the separation of metal catalysts after the reaction, the reaction mixture was first centrifuged (VWR Microstar 12) at 8200 × *g* for 10 min followed by syringe filtration. Syringe filter with a diameter size of 45 µm is made of PTFE (MULTOCLEAR-13, Chromatographie Service GmbH). Prior to analytics, the solution was treated with 0.01 M K_3_PO_4_ solution to precipitate dissolved metal species due to their paramagnetic effects in NMR spectra. Separated solid catalysts were washed with Milli-Q water and dried at a vacuum furnace overnight at 50 °C.

### Pyruvate conversion reactions

Sodium pyruvate-2-^13^C (99 at% C) was purchased from Sigma-Aldrich. Pyruvate was used as the only carbon source. 1 M metal powder (same amount as CO_2_ fixation) was added to 3 ml of aqueous Na-pyruvate solution (1 mg/ml) and the reaction was carried out at room temperature for different time intervals: 1 h, 24 h, 72 h, and 168 h. Initial pH of all reactions was 5 (due to the slightly acidic nature of pyruvate solution), except the studies wherein the effect of initial pH was investigated. The pH of the reaction solution was measured with both pH indicator strips (1.09526.0003, Universal indicator, Merck) and the Metrohm 848 Titrino Plus with a pH probe. For the reactions at pH 7 and 9, KOH solution (0.1 M) was used to adjust the pH of the reaction solution. Prior to experiments, the solution was bubbled with Ar gas for 5 min to eliminate possible dissolved carbon-based gases in the solution. Then, the solution was transferred to two Eppendorf tubes with a volume 2 ml and the tubes were sealed with parafilm. Although the reaction solution was purged with Ar, the reactions took place under atmospheric conditions in sealed Eppendorf tubes. Therefore, trace amounts of oxygen might be expected to exist in the reaction environment.

Shaking experiments were carried out with IKA KS 130 basic laboratory shaker (IKA® Werke GmbH & Co. KG, Staufen, Germany) at 560 rpm. To detect the gaseous products, the same reactions were performed in a sealed autoclave made of Mo-Ni with PTFE inlets. For that, the reactor was filled with 2 bar Ar before the reaction and the reactions were performed at 25 °C. Prior to reactions, the reactor was purged 3 times with Ar gas (5 bar). The autoclave reactions were performed under static conditions. For the analysis of released gas products, gas chromatography (GC, Agilent Technologies 7820 A) was utilized. Quantification of CO_2_ was determined with one-point calibration. The thermal conductivity detector (TCD) was used to detect CO_2_ and H_2_ gases. After each reaction, the autoclave was directly connected to the GC for the gas phase product analysis.

### Control experiments

Since reaction autoclaves were made of Mo–Ni alloys, two sets of control experiments were carried out to detect the potential C-species might be coming from the bulky reactor system without the addition of any solid catalyst. For this purpose, the reactor was filled with 3 ml Milli-Q water and was pressurized to 25 bar with CO_2_ + H_2_ (3:2) mixture and the reaction was carried out for 24 h at 25 °C. The backgrounds of Milli-Q water and reactor were also checked by pressurizing 3 ml H_2_O with 25 bar Ar.

Since spent tea leaves were used as a template for the synthesis of Ni-Fe alloys, we also performed control experiments to exclude the presence of any C-species. For this purpose, the same amount of metal catalyst (1 M) was added into 3 ml Milli-Q water without any CO_2_ as reactant. The high-pressure reactor was purged and pressurized with 25 bar of Ar instead of CO_2_ + H_2_ mixture and the reaction was carried out for 24 h at 25 °C. In the pyruvate conversion part, the control reactions were performed under the same conditions without the addition of a metal catalyst to test whether pyruvate converts to targeted products without any metal catalyst.

Potential conversions of obtained products acetate and citramalate were also examined. To check acetate conversion, Na-^12^C-acetate (1 mg/ml) was added to 2-^13^C-pyruvate solution (1 mg/ml) with 1 M Fe catalyst. The control experiments of pyruvate and acetate reaction were also performed without the addition of a metal catalyst. Reactions were carried out for 1 h at 25 °C. Another reaction was performed with only ^12^C-acetate as an only carbon source with Fe catalyst. There was no change in the concentration of ^12^C-acetate with or without the catalyst after 1 h of shaking.

To check the released gas products from the decomposition of pyruvate, 3 ml of pyruvate solution (1.0 mg/ml) was transferred into autoclave without any metal catalyst and pressurized with Ar gas to 2 bar. After 1 h of reaction time, the autoclave was directly connected to GC for the gas analysis.

For the citramalate conversion experiment, potassium citramalate salt (>97%, Sigma-Aldrich) was used as reactant. 1 M metal catalyst was added to an aqueous solution of citramalate (1 mg/ml) and the reaction solution was shaken for 1 h at 25 °C. 1 M Ni_3_Fe or Fe was used as the metal catalyst in 3 ml H_2_O.

### Product analysis

For the analysis of CO_2_ fixation products, HPLC and ^1^H-NMR methods were used. NMR, ESI-MS, and HPLC methods were used to analyze pyruvate conversion reactions.

It is crucial to precipitate possible leached metal species prior to NMR analysis due to their paramagnetic effects. After CO_2_ reduction reactions, the reaction solution was treated with 0.01 M K_3_PO_4_. A total of 100 µL of 0.1 M K_3_PO_4_ solution was added to 1 ml reaction solution and it was mixed by a Vortex mixer. Later, the precipitated metal ions were separated via centrifugation at 12300 × *g* (VWR Micro Star 12) for 15 min. NMR spectra were acquired on either one of a Bruker AVANCE NEO or a Bruker Avance III spectrometer, both operating at a field of 14.1 T (^1^H Larmor frequency of 600 MHz) and equipped with cryogenically cooled BBO (observe) or TCI (inverse) probeheads, for highest sensitivity on direct-observation of ^13^C or ^1^H, respectively. All spectra were collected at 298 K in standard 5 mm-tubes containing sample volumes of about 550 µL. In ^1^H spectra, water-suppression at approx. 4.68 ppm was achieved using excitation sculpting^90^ combined with a perfect echo^91^ using the Bruker standard pulse-program zgesgppe or a modified version thereof including a low-power adiabatic ^13^C-decoupling during acquisition bi_p5m4sp_4sp.2. The latter enabled to suppress satellites at natural abundance and coupling due to ^13^C-enrichment. With these sequences, it was possible to reduce the water signal intensity by a factor on the order of 10,000 while preserving good phase properties. On the other hand, due to the excitation profile of this sequence and the relatively short recycling delay (3 s), the signal intensities can only be considered as semi-quantitative. With this approach, it is estimated that product amounts down to a few tens of micrograms could be detected in a few hours (512–1024 iterations). The use of isotopically ^13^C-labeled pyruvate at position 2 not only added support to the identification of new products but also allows the monitoring of the atom-specific transformation pathway from the substrate to products. For ^13^C-spectra, a simple one-pulse excitation with ^1^H-decoupling (Bruker pulse-program zgdc30, ^1^H-decoupling scheme waltz65). Typically, 4k–8k scans were acquired for a total experimental time of 1.5–3 h. For selected Ni_3_Fe sample,Heteronuclear Multiple Bond Correlation (HMBC) experiment (Bruker: hmbcetgpl3nd) with standard parameters was acquired to confirm correlations over multiple-bonds (nJHC) between specific ^1^H and ^13^C-signals. To preserve integrity of the samples, no reference standard was added; referencing was generally done on 2-^13^C-pyruvate (^1^H: methyl (2.36ppm), ^13^C: C-2 (208 ppm)).

The concentrations of products were analyzed by high-performance liquid chromatography (HPLC) (Shimadzu LC-2030). For detection of CO_2_ fixation products, Metacarb column (300 × 7.5 mm) coupled with refractive index (RI) detector was operated at 50 °C. The mobile phase consisted of 0.1% trifluoroacetic acid (TFA) at a flow rate of 0.8 ml/min. For pyruvate conversion to citramalate reactions, a 100 mm organic resin column with an 8 mm diameter was operated at 40 °C. The mobile phase consisted of 2 mM TFA at the flow rate of 1 ml/min. ESI-MS spectra were recorded with Q ExactiveTM Plus Orbitrap mass spectrometer (Thermo Scientific, Bremen, Germany).

## Supplementary information

Supplementary Information

## Data Availability

All data supporting the findings of this study are available in the main text (Figs. 1–5) and supplementary information. Additional relevant source data are available from corresponding author upon request.
