# Supplementary material for: Ambient temperature CO2 fixation to pyruvate and subsequently to citramalate over iron and nickel nanoparticles
Source: Nat Commun. 2023 Feb 2;14:570. doi: 10.1038/s41467-023-36088-w (PMC9894855; doi:10.1038/s41467-023-36088-w)
Supplement: Supplementary file 1 — Supplementary Information [file 41467_2023_36088_MOESM1_ESM.pdf]

SUPPLEMENTARY INFORMATION

Ambient Temperature CO_2 Fixation to Pyruvate and Subsequently to Citramalate over Iron and Nickel Nanoparticles

Tugce Beyazay[1], Kendra S. Belthle[1], Christophe Farès[1], Martina Preiner[2], Joseph Moran[3], William F. Martin[4]*, Harun Tüysüz[1]*

[1] Max-Planck-Institute für Kohlenforschung, Kaiser-Wilhelm-Platz 1, 45470, Mülheim an der Ruhr, Germany

[2] Faculty of Geosciences, Utrecht University, Department of Ocean Systems, Royal Netherlands Institute for Sea Research (NIOZ), The Netherlands

[3] Université de Strasbourg, CNRS, ISIS UMR 7006, F-67000 Strasbourg, France

[4] Institute of Molecular Evolution, University of Düsseldorf, 40225 Düsseldorf, Germany

*E-mail: tueysuez@kofo.mpg.de ; bill@hhu.de

TABLE OF CONTENTS

Supplementary Fig. 1. TEM micrographs of Ni (a), Ni_3Fe (b), and Fe (c). N_2-sorption isotherms with offset 50 cm^3/g (d). 4
Supplementary Fig. 2. TG-MS data from Ni_3Fe catalyst under synthetic air from room temperature to 900 ˚C to analyze possible carbon contamination. m18 (blue) is MS of H_2O and m 44 is CO_2. No CO or CO_2 formation was detected............... 4
Supplementary Fig. 3. SEM-EDX analysis of Ni_3Fe that shows Ni and Fe as main elements with some minor impurities........ 5
Supplementary Fig. 4. The reaction setup for CO_2 conversion reactions. The reactor is made of Mo-Ni alloy. A PTFE or Ti inlet was used to minimize the contact of the reactants with the reactor. 6
Supplementary Fig. 5. HPLC chromatograph of standard solutions of possible CO_2 reduction products obtained on the Metacarb column. 6
Supplementary Fig. 6. HPLC results of the reaction of CO_2 + H_2 mixture (3:2 ratio), zoomed image shows the products at low concentrations (a), and the reaction of only CO_2 (25 bar) in H_2O at 25 ˚C for 24h over Ni_3Fe catalyst (b)............................ 8
Supplementary Fig. 7. Comparison of reactions of CO_2+ H_2 (a) and only CO_2 (b) in H_2O with Ni_3Fe catalyst under 25 bar at 25 ˚C for 24h. Data are presented as mean values of two independent experiments. 8
Supplementary Fig. 8. ^{1}H-NMR result of the reaction with CO_2 + H_2 mixture (25 bar) in H_2O over Ni_3Fe catalyst for 24 h at 25 ˚C. 9
Supplementary Fig. 9. ^{1}H-NMR result of the reaction performed with only CO_2 in H_2O without any molecular H_2 over Ni_3Fe catalyst at 25 ˚C for 24 h. Differences before and after K_3PO_4 treatment are also shown. K_3PO_4 treatment was done to precipitate possible leached metal ions in the reaction solution due to their paramagnetic effect in NMR measurement. K_3PO_4 also prevents the possible chelating of products with leached ion species. Due to these reasons, formate is visible after K_3PO_4 treatment. 9
Supplementary Fig. 10. HPLC results of control reactions of only the catalyst with Ar gas under 25 bar (a), and the reactor without catalyst in the presence of with CO_2 and H_2 gas mixture in H_2O at 25 ˚C for 24 h (b). Negligible amounts of formate were observed in the absence of the metal catalyst...10
Supplementary Fig. 11. XRD diagrams of Ni_3Fe before and after the reaction at 25 ˚C for 24h with CO_2 and H_2 mixture in H_2O. $FeCO_3$ reference is added in order to show that there is no carbonate formation after the reaction under 25 bar CO_2 + H_2 mixture at 25 ˚C..10
Supplementary Fig. 12. ESI-MS spectrum of pyruvate conversion reaction over Ni_3Fe catalyst after 1 h (a). ^{13}C-labelled acetate (m/z-:60), pyruvate (m/z-:88), and citramalate (m/z-:149.04) were observed. No unlabeled product was observed. ESI-MS spectrum of pyruvate conversion reaction in the absence of metal catalyst for 1 h (b). 11
Supplementary Fig. 13. ^{13}C-NMR spectra of reactions performed in autoclave (a) and in shaker (b) at 25 ˚C for 1h. Product range is the same for both reactors. 11
Supplementary Fig. 14. ^{13}C-NMR spectra of different reaction times (15 min to 2 h) of pyruvate conversion (1.0 mg/ml) over Ni_3Fe catalyst at 25 ˚C (a) Full spectrum and (b) zoomed spectrum to better resolve the formation of parapyruvate and citramalate. Pyr: Pyruvate, ppy: parapyruvate, act: acetate, citm: citramalate. 12
Supplementary Fig. 15. ^{1}H, ^{13}C-HMBC (Heteronuclear multiple bond correlation) spectrum of 1.0 mg/ml pyruvate conversion over Ni_3Fe catalyst after 24 h at 25 ˚C, showing distinctive 2-bond heteronuclear correlations of citramalate....12
Supplementary Fig. 16. ^{13}C-NMR spectra of different reaction times (1 h to 168 h) of pyruvate conversion (1.0 mg/ml) over Ni_3Fe catalyst at 25 ˚C. Original version (a) and zoomed version (b) in order to show the formation of parapyruvate and citramalate is in the right side. 13
Supplementary Fig. 17. ESI-MS results of pyruvate conversion reactions over Ni_3Fe catalyst after 1h (a), 24h (b), and 168h (c) with 1.0 mg/ml pyruvate at pH 5. 14

Supplementary Fig. 18. HPLC chromatograph of dissolved CO_2 in DI water for the qualitative analysis of released CO_2 during the reaction (a), pyruvate conversion over Ni_3Fe catalysts after 168h in order to show formed CO_2 during the reaction (b). 14
Supplementary Fig. 19. ESI-MS spectra of reactions at pH values of 7 (a) and 9 (b) with 1.0 mg/ml pyruvate over Ni_3Fe catalyst at 25 °C for 1 h. The control reaction of pH 9 without any solid catalyst (c) is also shown. Labelled citramalate and parapyruvate were observed at both pH 7 and 9 with Ni_3Fe catalyst. 15
Supplementary Fig. 20. ^{13}C-NMR spectra of reactions at pH 7 (a) and 9 (b) over Ni_3Fe catalyst at 25 °C for 1 h. Control reaction without any solid catalyst (c) is also added. Citramalate was observed at both pH 7 and 9 with the metal catalyst. However, only pyruvate and dihydroxy propionate were observed without the addition of a metal catalyst after 1 h. 16
Supplementary Fig. 21. HPLC chromatograph of pyruvate conversion over Ni_3Fe catalyst after 1h (a), and the result from the reaction continued after taking out the solid catalyst for 24h. (b) No significant changes have been observed without solid catalyst that indicates that the catalytic activity of the leached metal species are negligible. 17
Supplementary Fig. 22. ^{13}C-NMR spectra of the reaction between ^{12}C-acetate and ^{13}C-pyruvate (a) and only ^{13}C-pyruvate (b) over Ni_3Fe catalyst at 25 °C for 24h. There was no detection of single-carbon-labeled citramalate........17
Supplementary Fig. 23. ESI-MS spectrum of ^{13}C-pyruvate and 12C-acetate reaction with Ni_3Fe catalyst. While two carbon labelled citramalate was detected at m/z-: 149.037 in zoom area, one carbon-labelled citramalate was not detected at m/z-: 148........18
Supplementary Fig. 24. HPLC results of ^{13}C-pyruvate and ^{12}C-acetate reaction over Ni_3Fe catalyst after 1h (a). Control reaction is shown (b) without the addition of the catalyst under the same reaction conditions. 18
Supplementary Fig. 25. HPLC results of ^{12}C-acetate conversion reaction with Ni_3Fe catalyst (a) and the control experiment without the catalyst (b) after 1h of shaking. There was no noticeable conversion of acetate with or without the metal catalyst........19
Supplementary Fig. 26. Gas chromatographm of CO_2 standard (a). The CO_2 peak was observed at 4.78 min. Pyruvate was shaken for 1 h without any metal catalyst as a control reaction (b). Negligible amount of CO_2 compared to reactions with metal catalysts was observed. For the analysis of CO_2, Thermal Conductivity Detector (TCD) was used. 19
Supplementary Fig. 27. Gas products obtained from pyruvate conversion (1.0 mg/ml) over Fe catalyst during 1h (a) and 168h (b) of reaction times at 25 °C under 2 bar Ar. The amount of CO_2 after 168h was four times higher than 1h of the reaction time with Fe^0 catalyst. 20
Supplementary Fig. 28. Gas products obtained from pyruvate conversion (1.0 mg/ml) over Ni (a) and Fe catalyst (b) during 168h of reaction times at 25 °C. H_2 gas was observed in addition to CO_2 gas with Ni metal catalyst after 168 h under 2 bar Ar. The amount of CO_2 gas after 168 h was higher with Fe^0 catalyst compared to Ni^0........20
Supplementary Fig. 29. HPLC chromatograph of pyruvate conversion over Ni^0 (a), Fe^0 (b), and Ni_3Fe (c) catalysts after 168 h that shows also formation of dissolved CO_2 during the reaction. The amount of dissolved CO_2 after 168 h pyruvate conversion reaction was highest over Fe^0 catalyst and lowest over Ni^0 catalyst. 21
Supplementary Fig. 30. HPLC results of pyruvate conversion (1.0 mg/ml) after 1h over Fe nanoparticles (a) and Fe bulk catalyst (b). Higher conversion of pyruvate was observed over nanoparticulate Fe catalyst after 1h. ND : Not detected ...21
Supplementary Fig. 31. XPS spectra of Ni 2p before the reaction (a), after 168h of the reaction time (b), and Fe 2p before the reaction (c), after the reaction time of 168h (d). After 168h of the reaction time, metallic Fe and Ni were disappeared. 22
Supplementary Fig. 32. HPLC chromatographs of citramalate conversion reactions without any metal catalyst (a) and with Ni_3Fe catalyst after 1h (b). Initial concentration of citramalate is 1.0 mg/ml. There was not any conversion without the addition of a metal catalyst. 23
Supplementary Fig. 33. HPLC chromatographs of citramalate conversion over Fe^0 catalyst after 30 min (a) and 1 h (b). After 30 min of the reaction, some intermediate products, such as formate, acetate, and lactate, were observed. 23

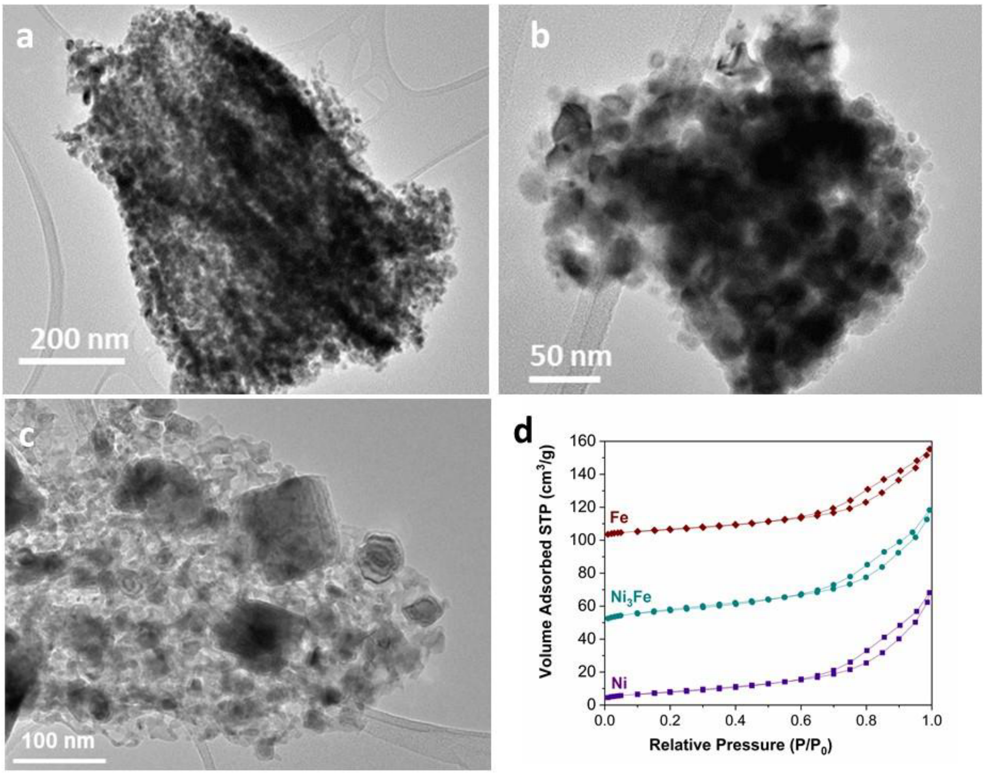

Supplementary Fig. 1. TEM micrographs of Ni (a), Ni_3Fe (b), and Fe (c). N_2-sorption isotherms with offset 50 cm^3/g (d).

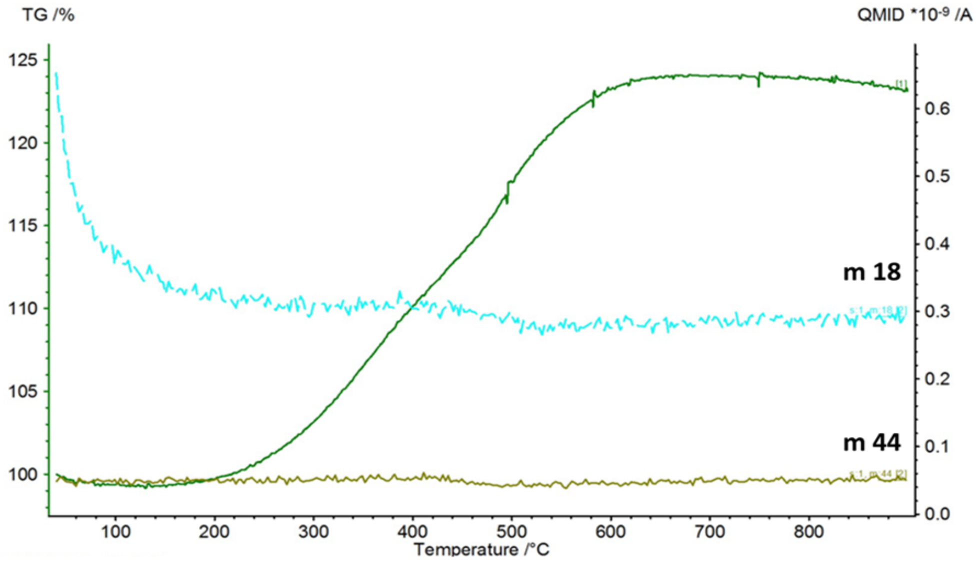

Supplementary Fig. 2. TG-MS data from Ni_3Fe catalyst under synthetic air from room temperature to 900 ˚C to analyze possible carbon contamination. m18 (blue) is MS of H_2O and m 44 is CO_2. No CO or CO_2 formation was detected.

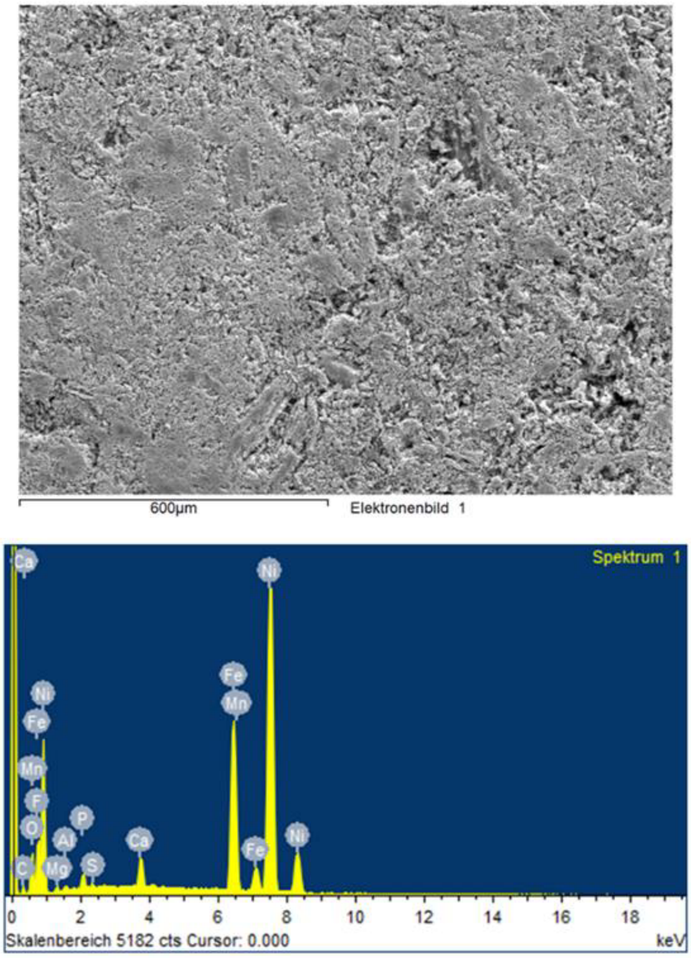

Supplementary Fig. 3. SEM-EDX analysis of Ni_3Fe that shows Ni and Fe as main elements with some minor impurities.

Supplementary Table 1. Bulk elemental analysis of Ni_3Fe catalyst

Element	Mass%	Mass% Sigma	Atom%
O K	7.32	0.26	21.12
Mg K	0.92	0.12	1.75
Al K	0.50	0.09	0.86
P K	1.36	0.08	2.03
S K	0.27	0.06	0.39
Ca K	1.91	0.06	2.20
Mn K	0.13	0.06	0.11
Fe K	21.64	0.20	17.88
Ni K	64.85	0.35	50.98
Total	100.00		

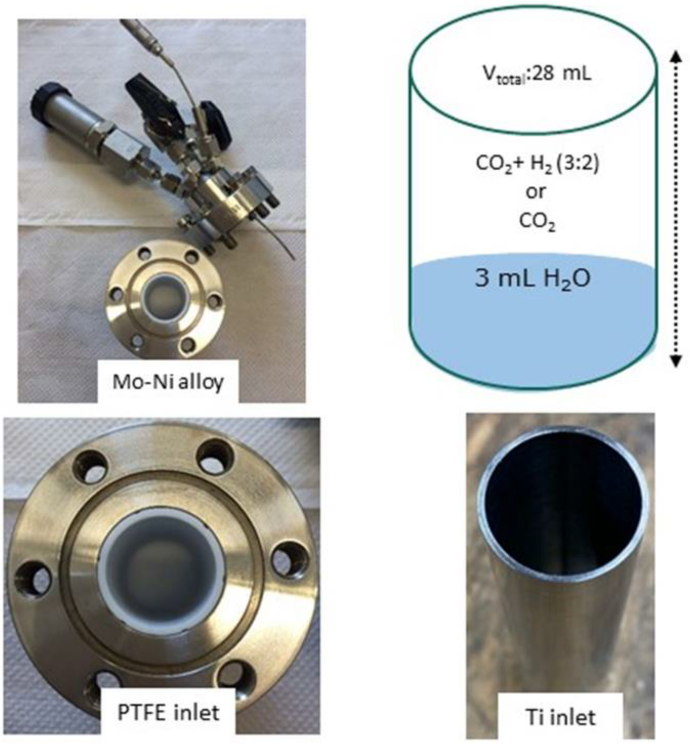

Supplementary Fig. 4. The reaction setup for CO_2 conversion reactions. The reactor is made of Mo-Ni alloy. A PTFE or Ti inlet was used to minimize the contact of the reactants with the reactor.

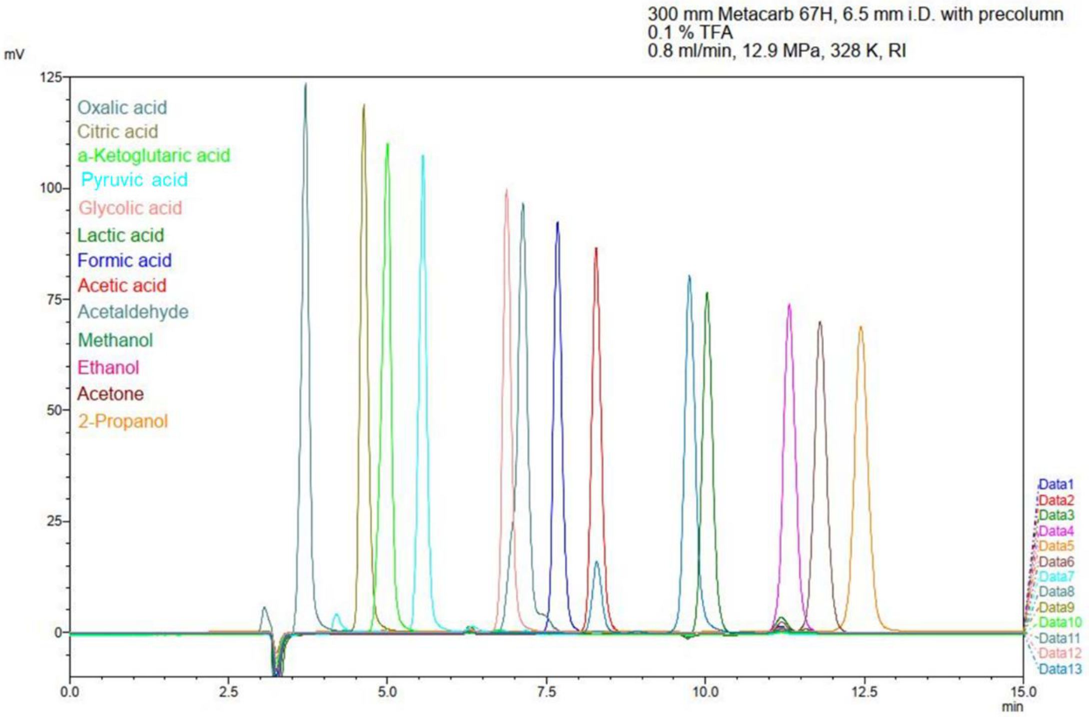

Supplementary Fig. 5. HPLC chromatograph of standard solutions of possible CO_2 reduction products obtained on the Metacarb column.

Supplementary Table 2. Retention time (RT) and refractive index (RI) of possible CO_2 reduction products on Metacarb.

Substance	Molecule	Retention time (min)	Refractive Index
Oxalic Acid	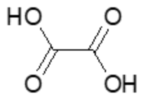	3.82	81366
Citric Acid	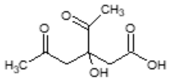	4.64	76352
Pyruvic Acid	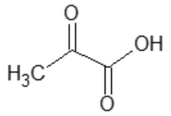	5.78	63607
Glycolic Acid	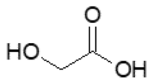	6.96	57999
Lactic Acid	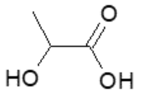	7.24	-
Formic Acid	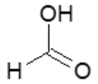	7.81	33371
Acetic Acid	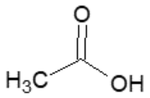	8.48	42251
Acetaldehyde	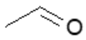	10.08	-
Methanol	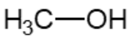	10.45	10732
Ethanol	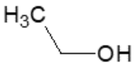	11.87	35371
Acetone	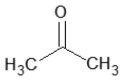	12.31	41946
2-Propanol	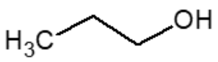	13.12	53298

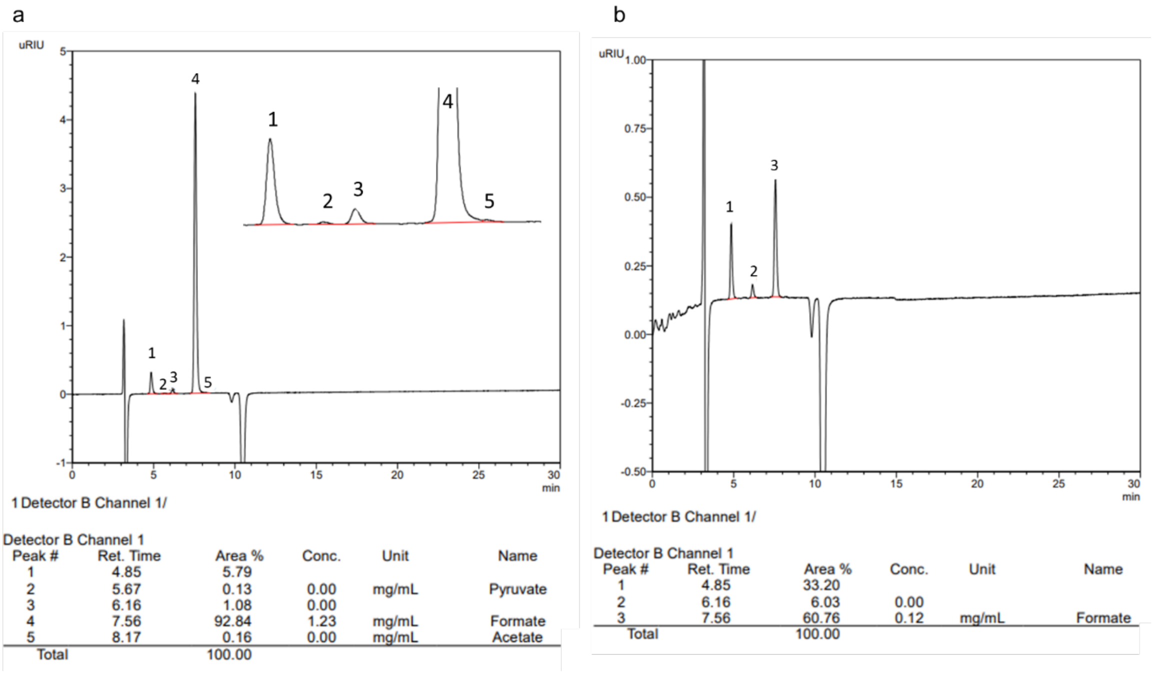

(a)

Peak #	Ret. Time	Area %	Conc.	Unit	Name
1	4.85	5.79			
2	5.67	0.13	0.00	mg/mL	Pyruvate
3	6.16	1.08	0.00		
4	7.56	92.84	1.23	mg/mL	Formate
5	8.17	0.16	0.00	mg/mL	Acetate
Total		100.00			

(b)

Peak #	Ret. Time	Area %	Conc.	Unit	Name
1	4.85	33.20			
2	6.16	6.03	0.00		
3	7.56	60.76	0.12	mg/mL	Formate
Total		100.00			

Supplementary Fig. 6. HPLC results of the reaction of CO_2 + H_2 mixture (3:2 ratio), zoomed image shows the products at low concentrations (peaks 1 and 3 correspond to water impurities, 2 is pyruvate, 4 is formate, and 5 is acetate) (a), and the reaction of only CO_2 (25 bar) in H_2O at 25 ˚C for 24h over Ni_3Fe catalyst (peaks 1 and 2 are water impurities and peak 3 is formate) (b).

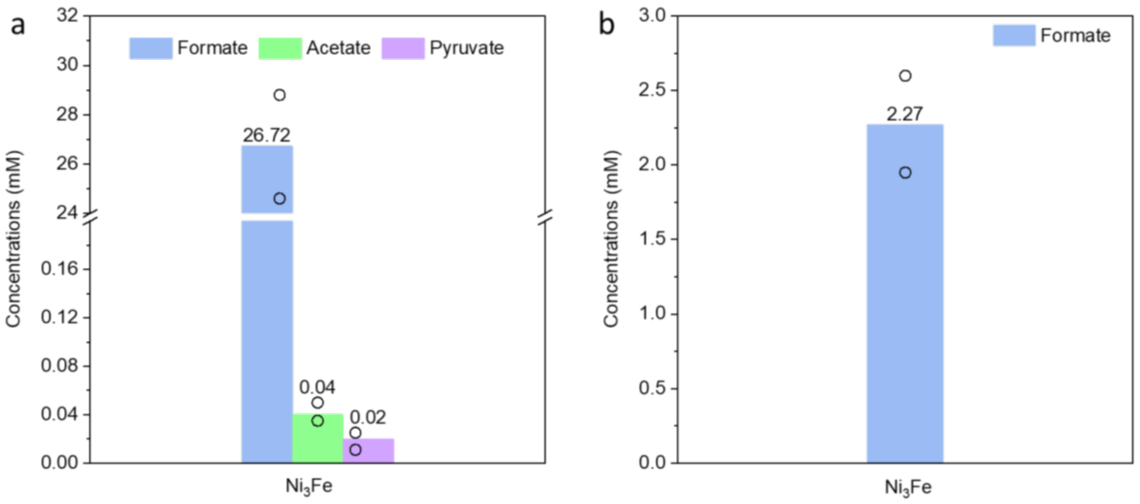

Supplementary Fig. 7. Comparison of reactions of CO_2+ H_2 (a) and only CO_2 (b) in H_2O with Ni_3Fe catalyst under 25 bar at 25 ˚C for 24h. Data are presented as mean values of two independent experiments.

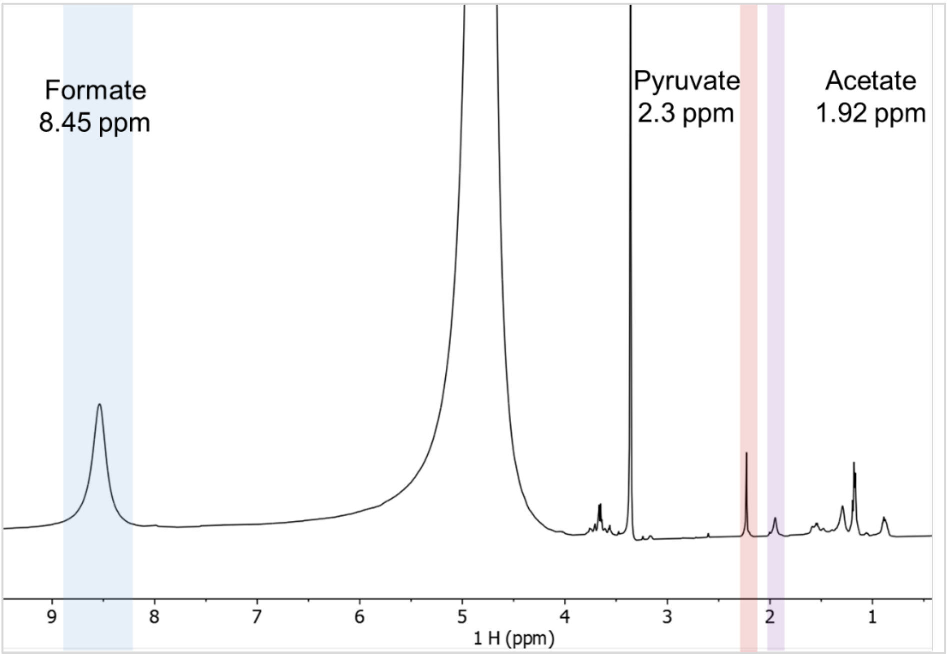

Supplementary Fig. 8. ^{1}H-NMR result of the reaction with $CO_2 + H_2$ mixture (25 bar) in H_2O over Ni_3Fe catalyst for 24 h at 25 ˚C.

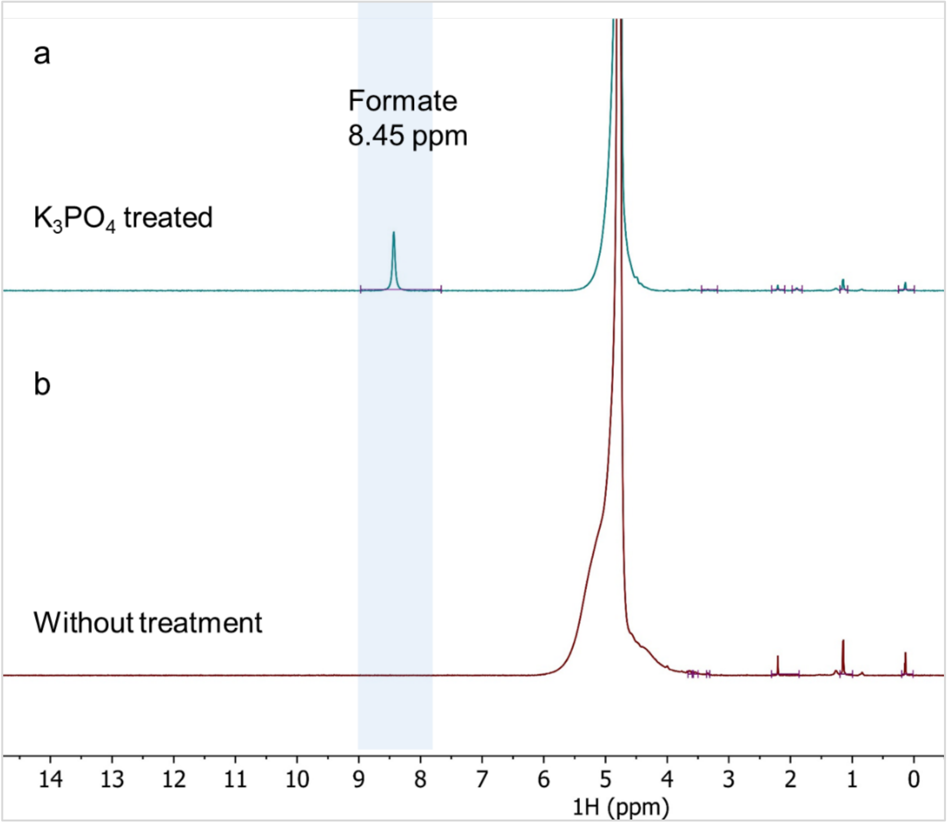

Supplementary Fig. 9. ^{1}H-NMR result of the reaction performed with only CO_2 in H_2O without any molecular H_2 over Ni_3Fe catalyst at 25 ˚C for 24 h with (a) or without (b) K_3PO_4 treatment. K_3PO_4 treatment was done to precipitate possible leached metal ions in the reaction solution due to their paramagnetic effect in NMR measurement. K_3PO_4 also prevents the possible chelating of products with leached ion species. Due to these reasons, formate is visible after K_3PO_4 treatment.

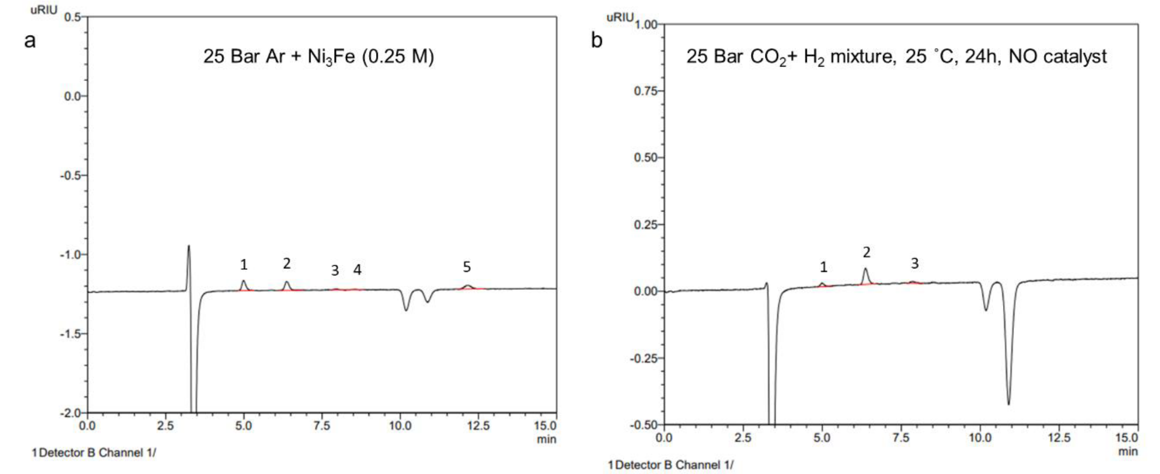

Detector B Channel 1

Peak #	Ret. Time	Area %	Conc.	Unit	Name
1	4.99	37.43	0.00		
2	6.37	36.28	0.00		
3	7.95	3.52	0.00	mg/mL	Formate
4	8.55	-0.03	0.00	mg/mL	Acetate
5	12.16	22.79	0.01	mg/mL	Aceton
Total		100.00			

Detector B Channel 1

Peak #	Ret. Time	Area %	Conc.	Unit	Name
1	5.00	14.18	0.00		
2	6.37	76.05	0.00		
3	7.86	9.77	0.00	mg/mL	Formate
Total		100.00			

Supplementary Fig. 10. HPLC results of control reactions of only the catalyst with Ar gas under 25 bar (peaks 1 and 2 are water impurities and peaks 3, 4, and 5 are formate, acetate, and acetone, respectively) (a), and the reactor without catalyst in the presence of CO_2 and H_2 gas mixture in H_2O at 25 ˚C for 24 h (peaks 1 and 2 are water impurities, peak 3 is formate) (b). Negligible amounts of formate were observed in the absence of the metal catalyst.

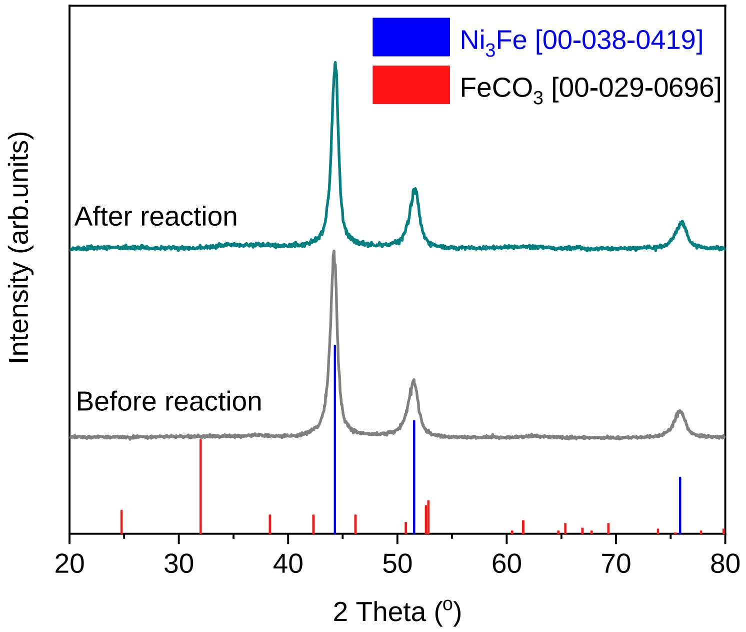

Supplementary Fig. 11. XRD diagrams of Ni_3Fe before and after the reaction at 25 ˚C for 24h with CO_2 and H_2 mixture in H_2O. $FeCO_3$ reference is added in order to show that there is no carbonate formation after the reaction under 25 bar CO_2 + H_2 mixture at 25 ˚C.

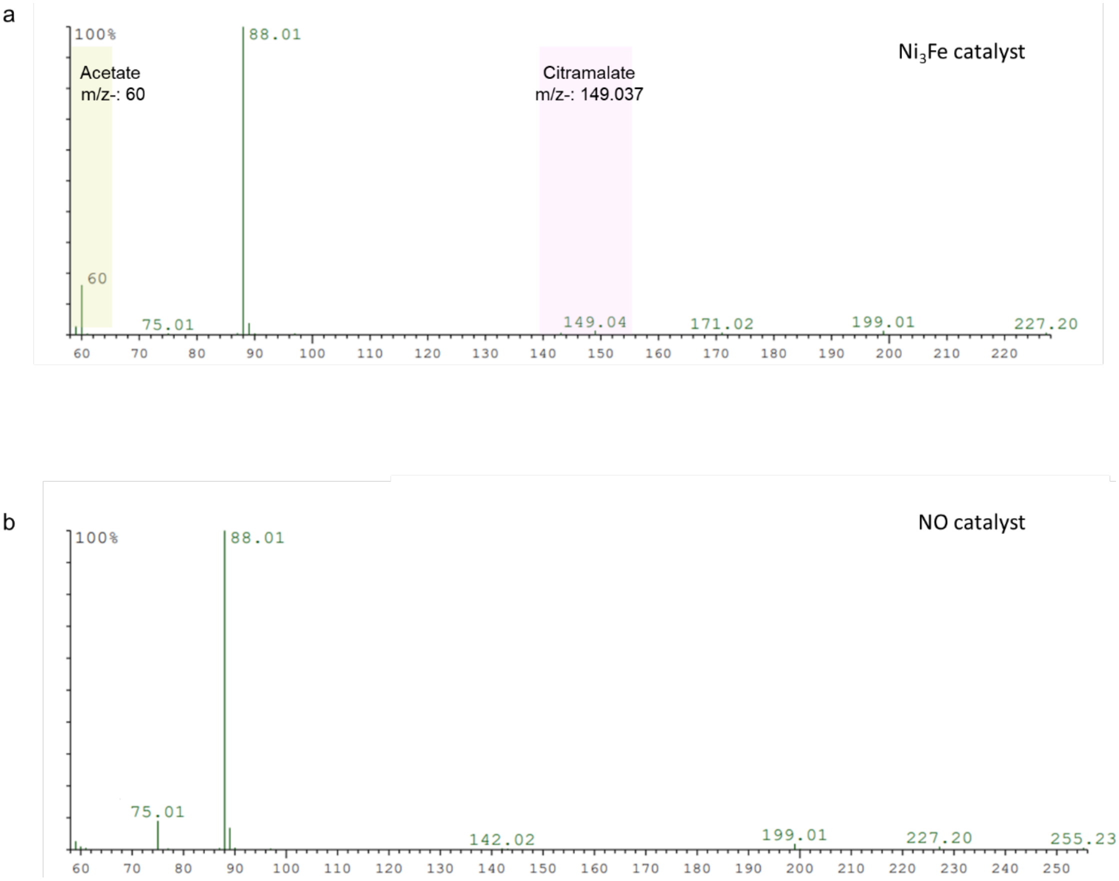

Supplementary Fig. 12. ESI-MS spectrum of pyruvate conversion reaction over Ni_3Fe catalyst after 1 h (a). ^{13}C-labelled acetate (m/z-:60), pyruvate (m/z-:88), and citramalate (m/z-:149.04) were observed. No unlabeled product was observed. ESI-MS spectrum of pyruvate conversion reaction in the absence of metal catalyst for 1 h (b). No citramalate was observed.

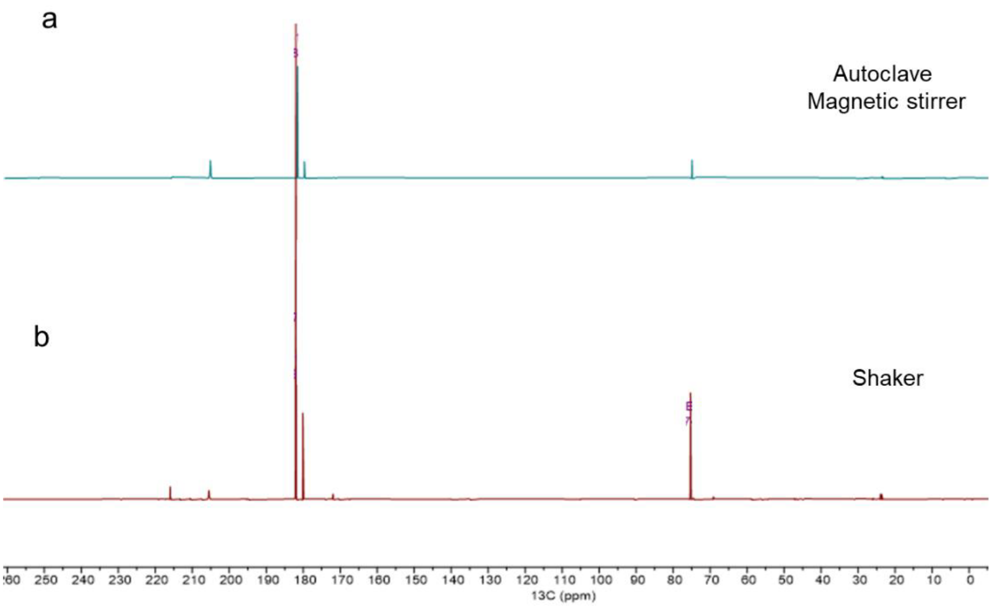

Supplementary Fig. 13. ^{13}C-NMR spectra of reactions performed in an autoclave (a) and in shaker (b) with 1.0 mg/ml pyruvate at 25 ˚C for 1h. Product range is the same for both reactors.

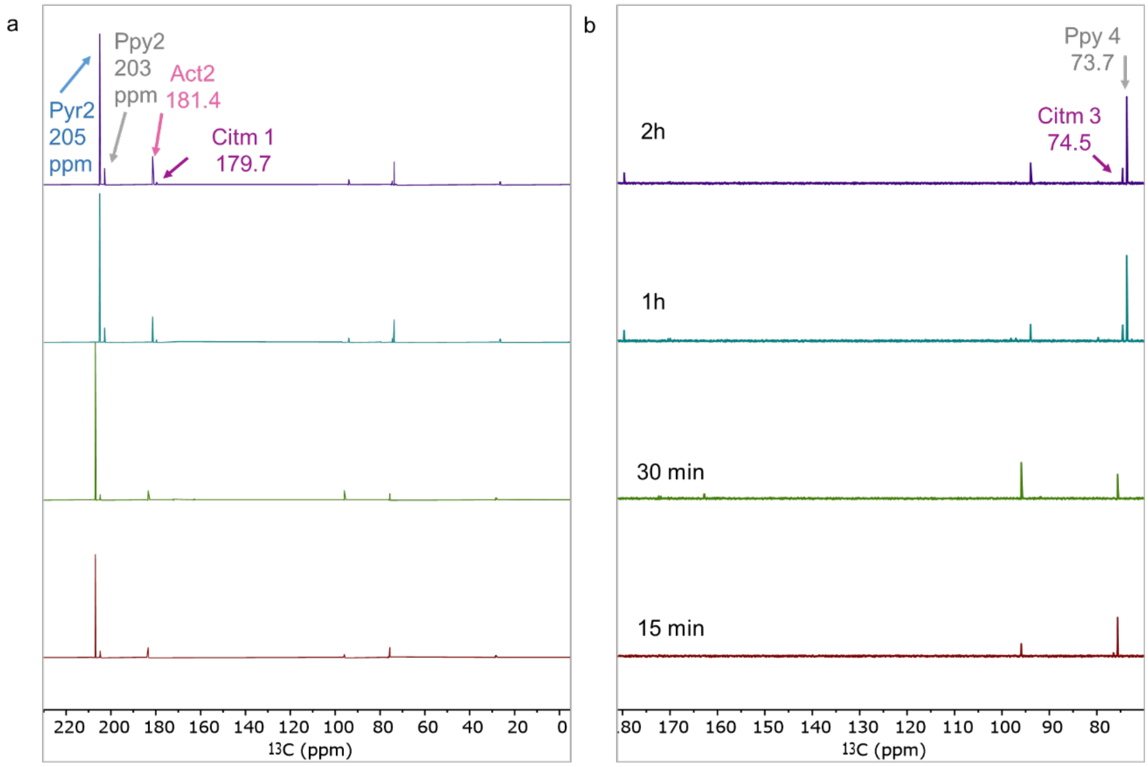

Supplementary Fig. 14. ^{13}C-NMR spectra of different reaction times (15 min to 2 h) of pyruvate conversion (1.0 mg/ml) over Ni_3Fe catalyst at 25 ˚C. (a) Full spectrum and (b) zoomed spectrum to better resolve the formation of parapyruvate and citramalate. Pyr: Pyruvate, ppy: parapyruvate, act: acetate, citm: citramalate.

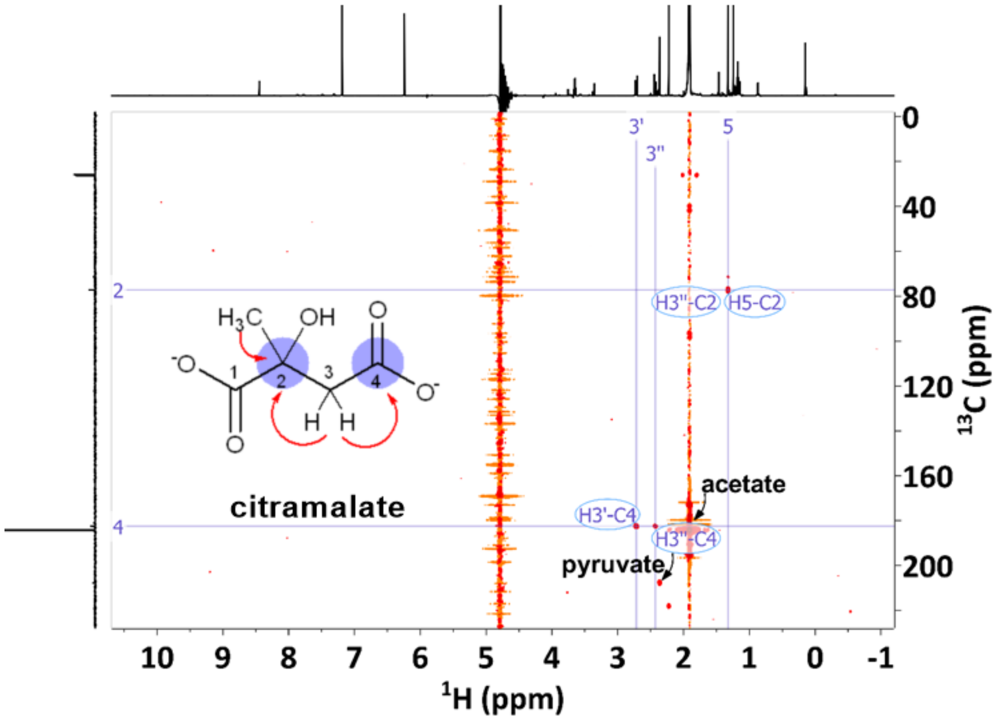

Supplementary Fig. 15. ^{1}H, ^{13}C-HMBC (Heteronuclear multiple bond correlation) spectrum of 1.0 mg/ml pyruvate conversion over Ni_3Fe catalyst after 24 h at 25 ˚C, showing distinctive 2-bond heteronuclear correlations of citramalate.

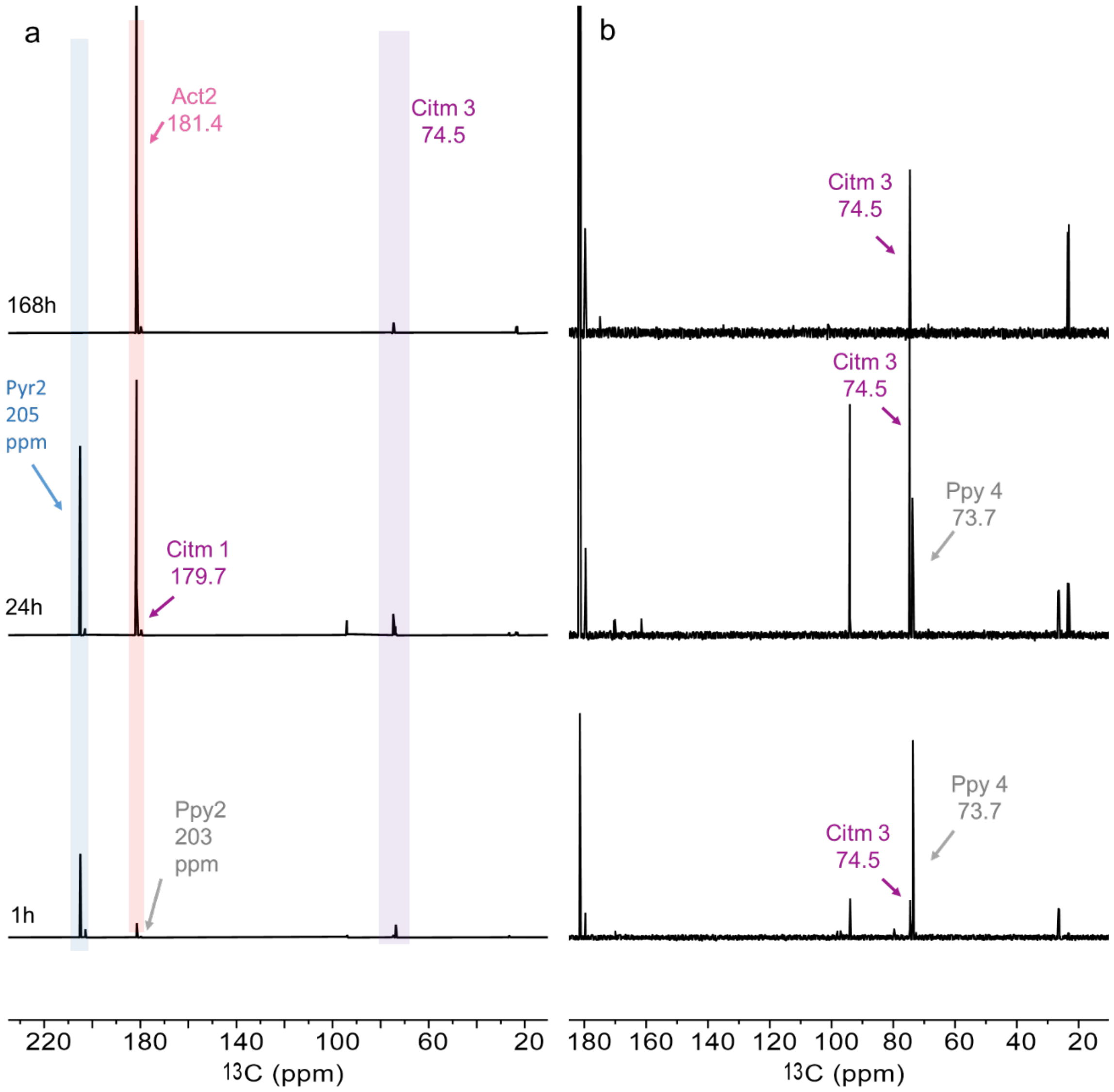

Supplementary Fig. 16. ^{13}C-NMR spectra of different reaction times (1 h to 168 h) of pyruvate conversion (1.0 mg/ml) over Ni_3Fe catalyst at 25 °C. Original version (a) and zoomed version (b) in order to show the formation of parapyruvate and citramalate is in the right side. Pyr: Pyruvate, ppy: parapyruvate, act: acetate, citm: citramalate.

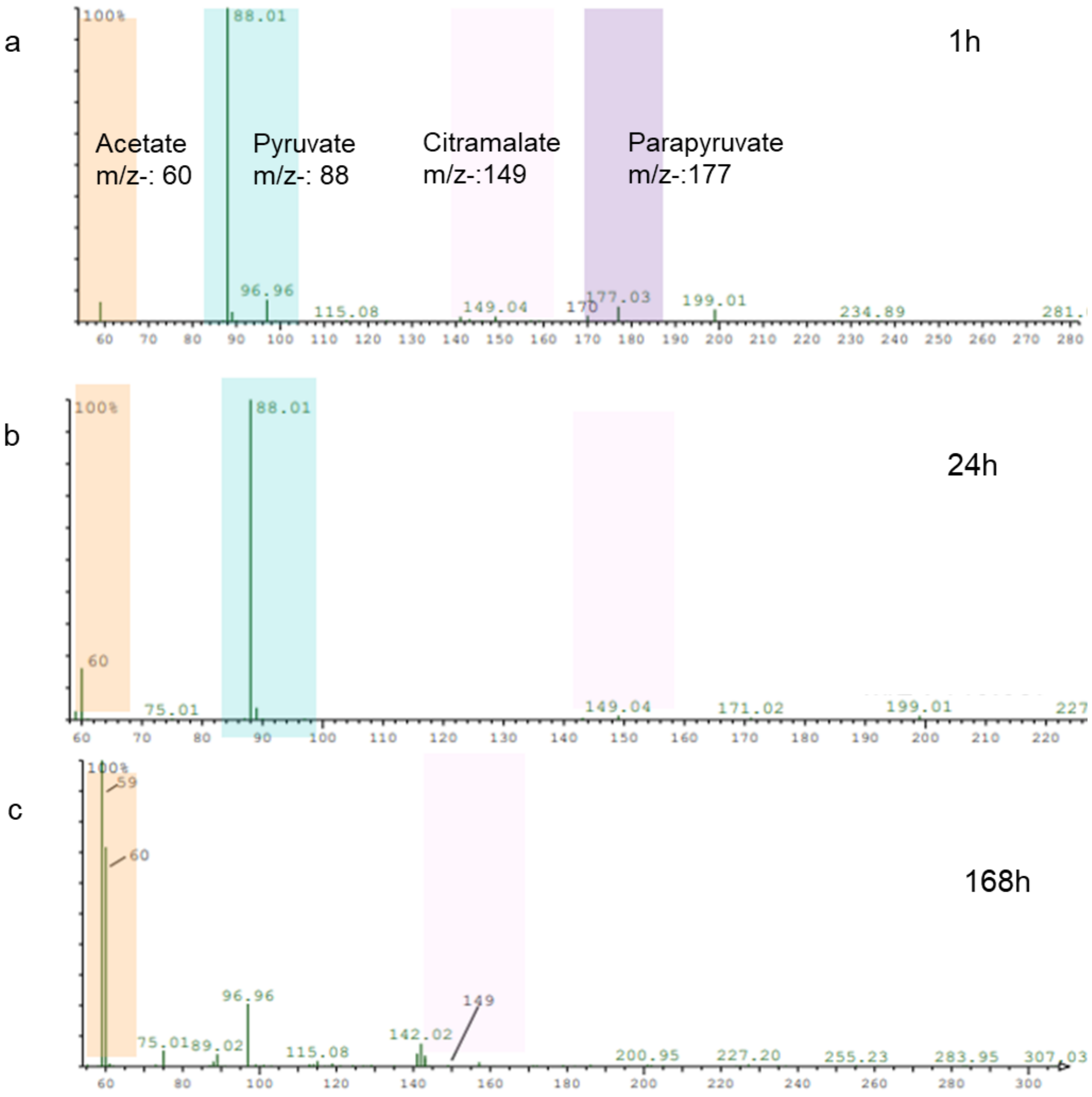

Supplementary Fig. 17. ESI-MS results of pyruvate conversion reactions over Ni_3Fe catalyst after 1h (a), 24h (b), and 168h (c) with 1.0 mg/ml pyruvate at pH 5 at 25 ˚C.

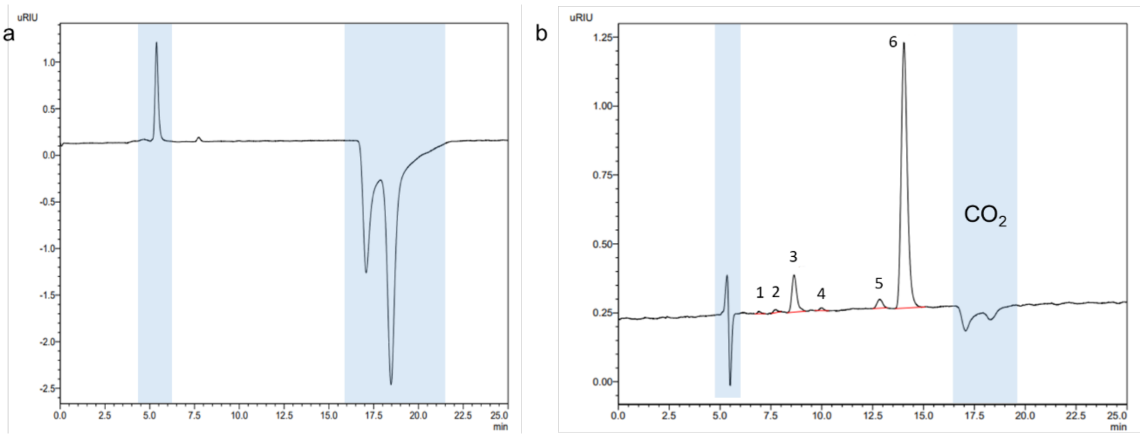

Supplementary Fig. 18. HPLC chromatograph of dissolved CO_2 in DI water for the qualitative analysis of released CO_2 during the reaction (a), pyruvate conversion over Ni_3Fe catalyst after 168h at 25 ˚C in order to show formed CO_2 during the reaction (b). Peaks 1, 3, and 6 correspond to pyruvate, citramalate, and acetate, respectively and remaining small peaks correspond to possible impurities after the decomposition reaction.

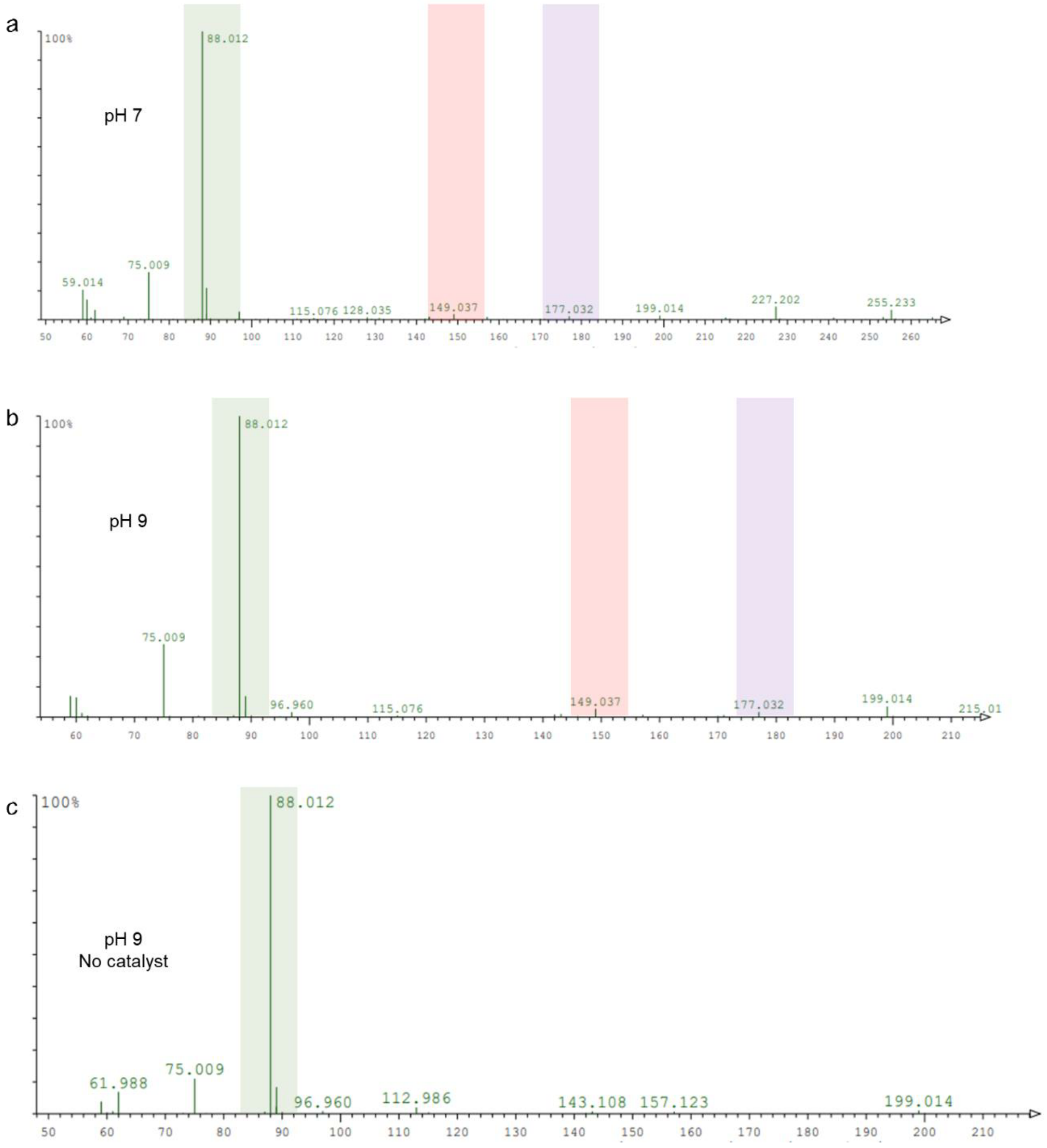

Supplementary Fig. 19. ESI-MS spectra of reactions at pH values of 7 (a) and 9 (b) with 1.0 mg/ml pyruvate over Ni_3Fe catalyst at 25 ˚C for 1 h. The control reaction of pH 9 without any solid catalyst (c) is also shown. Labelled citramalate and parapyruvate were observed at both pH 7 and 9 with Ni_3Fe catalyst.

Supplementary Table 3. ICP-OES results of the reaction solutions after the reaction time of 1 h and 2 h with 1.0 mg/ml pyruvate over Ni_3Fe catalyst. Total concentration of Fe ion was obtained by the average of characteristic wavelengths of Fe ions, 238.204 nm and 259.940 nm. Total concentration of Ni ion in the solution was obtained by the average of the characteristic wavelengths at 221.648 nm and 231.604 nm.

Reaction Time	Fe 238.204 nm (µg/ml)	Fe 259.940 nm (µg/ml)	Fe Total Concentration (µg/ml)	Ni 221.648 nm (µg/ml)	Ni 231.604 nm (µg/ml)	Ni Total Concentration (µg/ml)
1h	0.1833	0.1833	0.183	0.0433	0.0445	0.044
2h	0.211	0.214	0.213	0.0663	0.0620	0.064

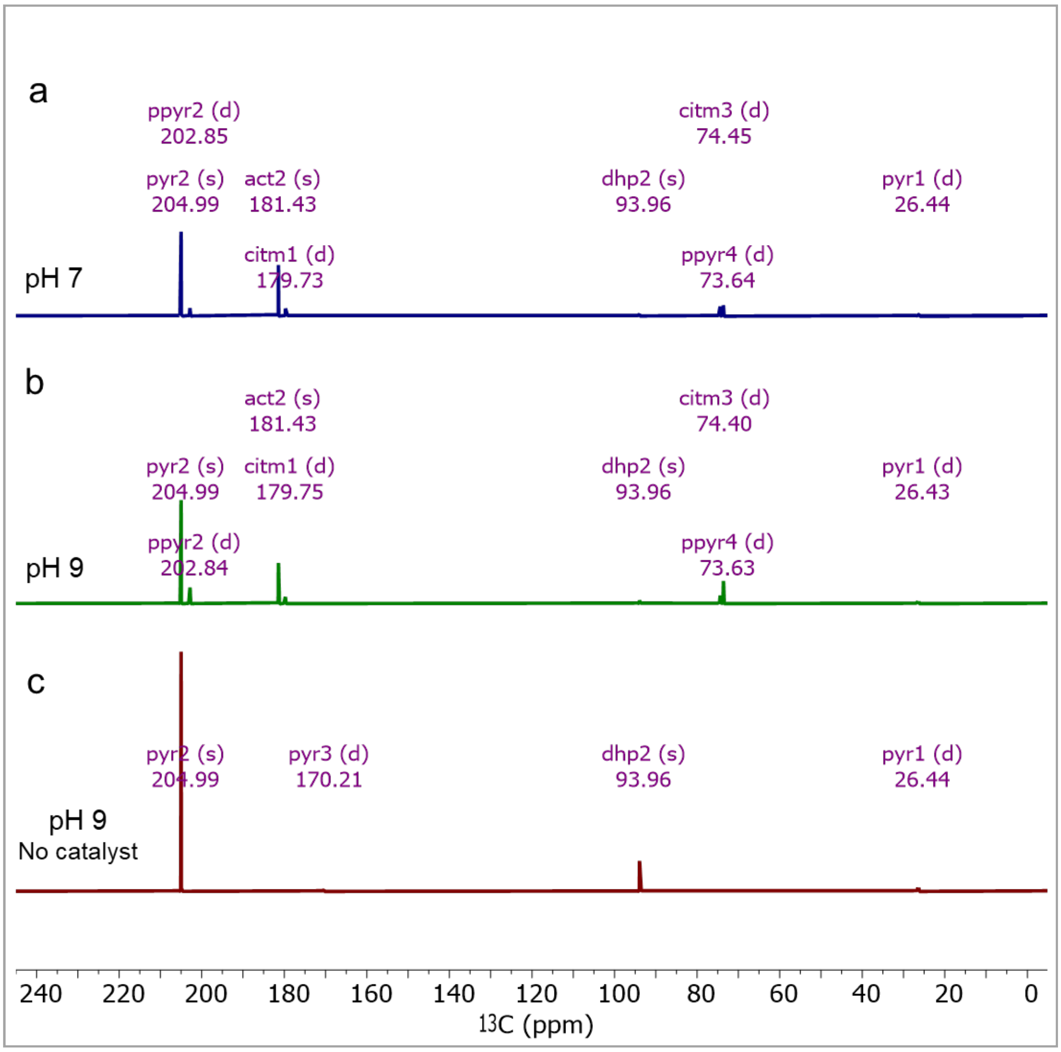

Supplementary Fig. 20. ^{13}C-NMR spectra of reactions at pH 7 (a) and 9 (b) over Ni_3Fe catalyst at 25 °C for 1 h. Control reaction without any solid catalyst (c) is also added. Citramalate was observed at both pH 7 and 9 with the metal catalyst. However, only pyruvate and dihydroxy propionate were observed without the addition of a metal catalyst after 1 h. Pyr: Pyruvate, ppy: parapyruvate, act: acetate, citm: citramalate, dhp: dihydroxypropionate.

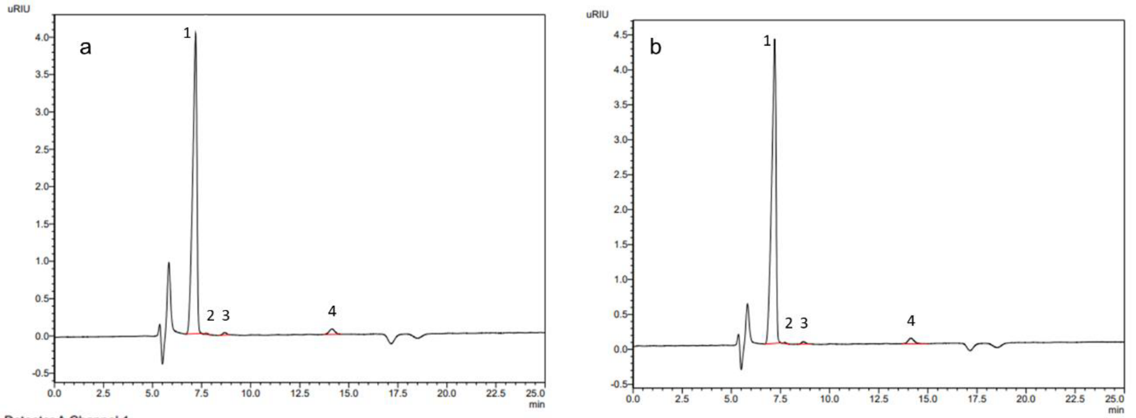

Detector A Channel 1 (a)

Peak #	Ret. Time	Area %	Conc.	Units	Name
1	7.19	96.75	0.94	mg/mL	Pyruvate
2	7.73	0.25	0.00		
3	8.68	0.73	0.01	mg/mL	Citramalate
4	14.14	2.27	0.04	mg/mL	Acetate
Total		100.00			

Detector A Channel 1 (b)

Peak #	Ret. Time	Area %	Conc.	Units	Name
1	7.21	96.68	1.05	mg/mL	Pyruvate
2	7.72	0.19	0.00		
3	8.67	0.69	0.01	mg/mL	Citramalate
4	14.14	2.44	0.05	mg/mL	Acetate
Total		100.00			

Supplementary Fig. 21. HPLC chromatograph of pyruvate conversion over Ni_3Fe catalyst after 1h (a), and the result from the reaction continued after taking out the solid catalyst for 24h (b). Peak 1 is pyruvate, 2 is water impurity, 3 is citramalate, and 4 is acetate in both chromatographs. No significant changes have been observed without solid catalyst that indicates that the catalytic activity of the leached metal species are negligible.

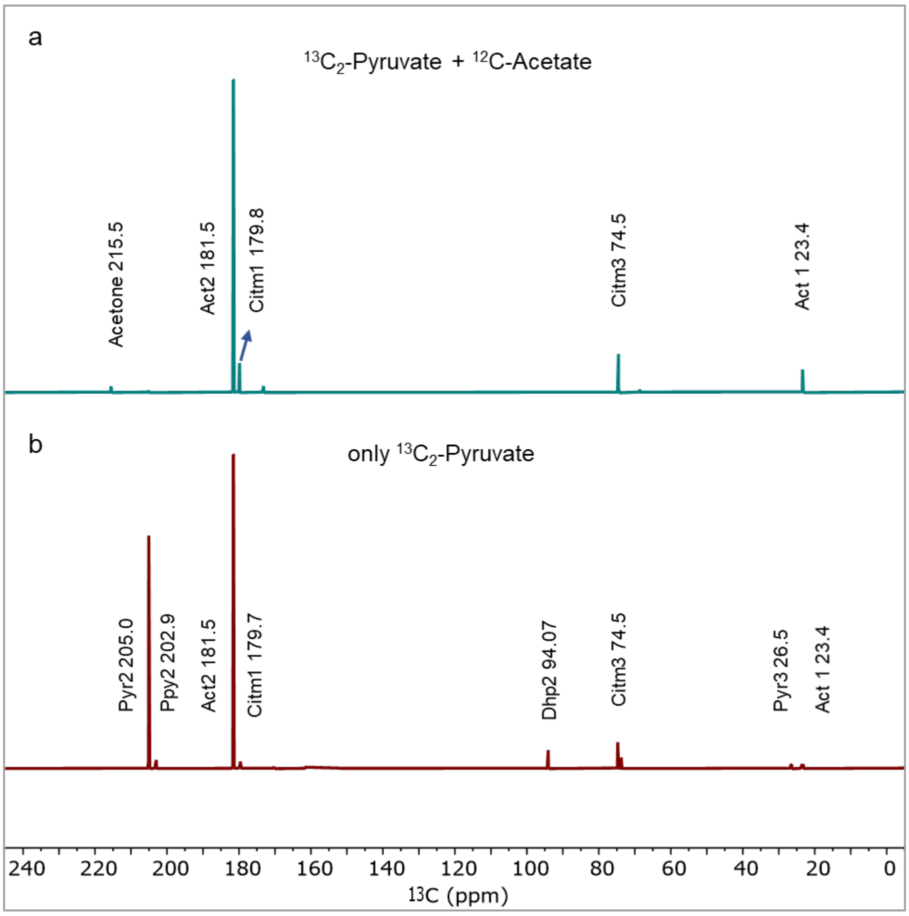

Supplementary Fig. 22. ^{13}C-NMR spectra of the reaction between ^{12}C-acetate and ^{13}C-pyruvate (a) and only ^{13}C-pyruvate (b) over Ni_3Fe catalyst at 25 °C for 24h. There was no detection of single-carbon-labeled citramalate. Pyr: Pyruvate, ppy: parapyruvate, act: acetate, citm: citramalate.

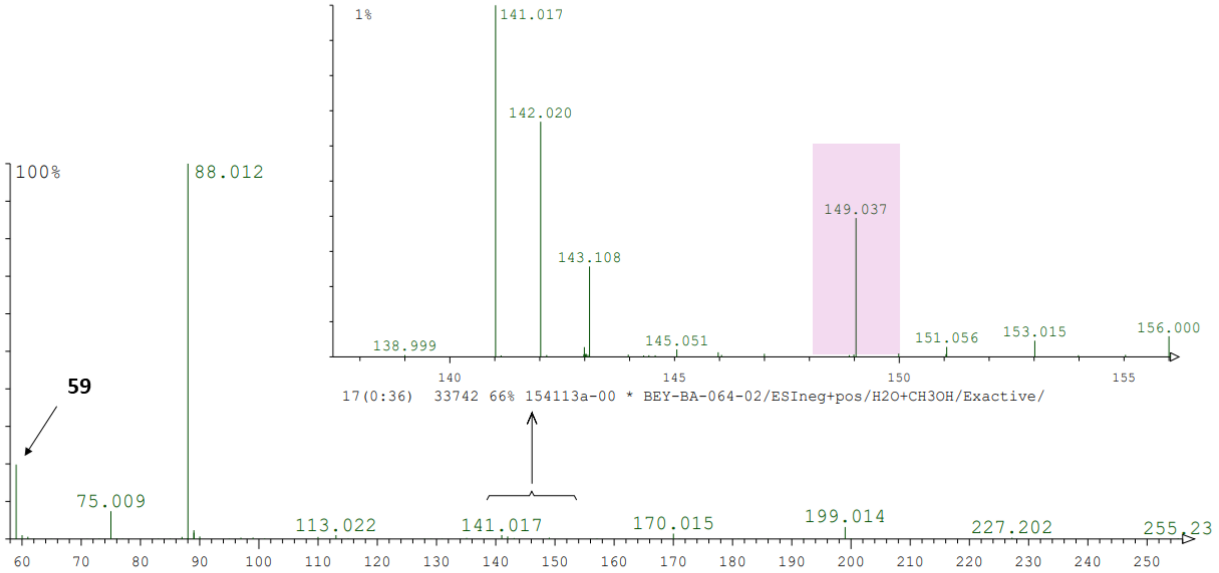

Supplementary Fig. 23. ESI-MS spectrum of ^{13}C-pyruvate and ^{12}C-acetate reaction with Ni_3Fe catalyst. While two carbon labelled citramalate was detected at m/z-: 149.037 in zoom area, one carbon-labelled citramalate was not detected at m/z-: 148.

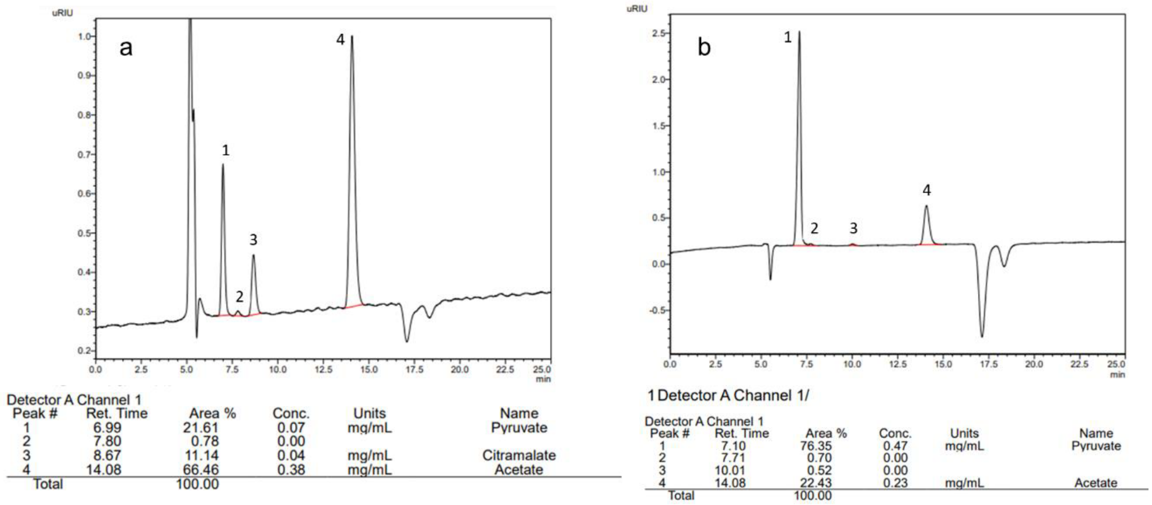

Supplementary Fig. 24. HPLC results of ^{13}C-pyruvate and ^{12}C-acetate reaction over Ni_3Fe catalyst after 1h (peak 1 is pyruvate, 2 is water impurities, 3 and 4 are citramalate and acetate, respectively) (a). Control reaction is shown (b) without the addition of the catalyst under the same reaction conditions (peak 1 is pyruvate, 2 and 3 are water impurities, 4 is acetate).

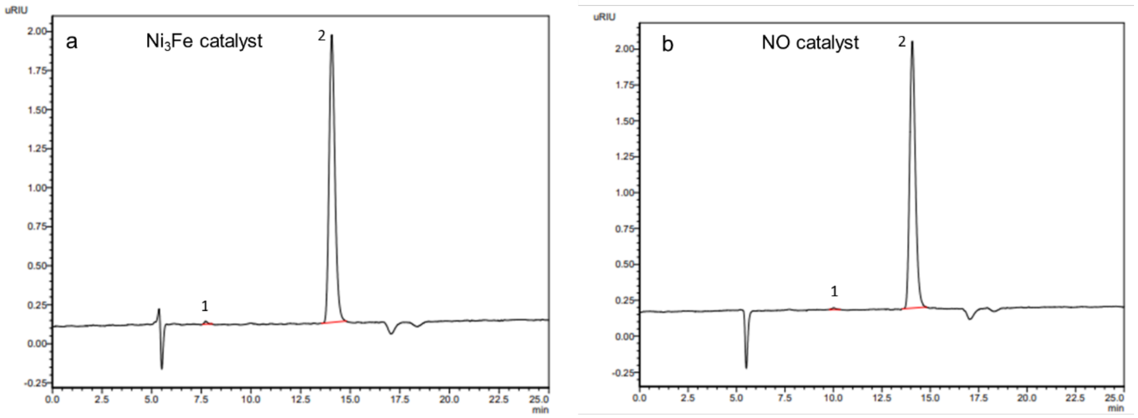

Supplementary Fig. 25. HPLC results of ^{12}C-acetate conversion reaction with Ni_3Fe catalyst (a) and the control experiment without the catalyst (b) after 1h of shaking. There was no noticeable conversion of acetate with or without the metal catalyst (peak 1 is water impurities, 2 is acetate).

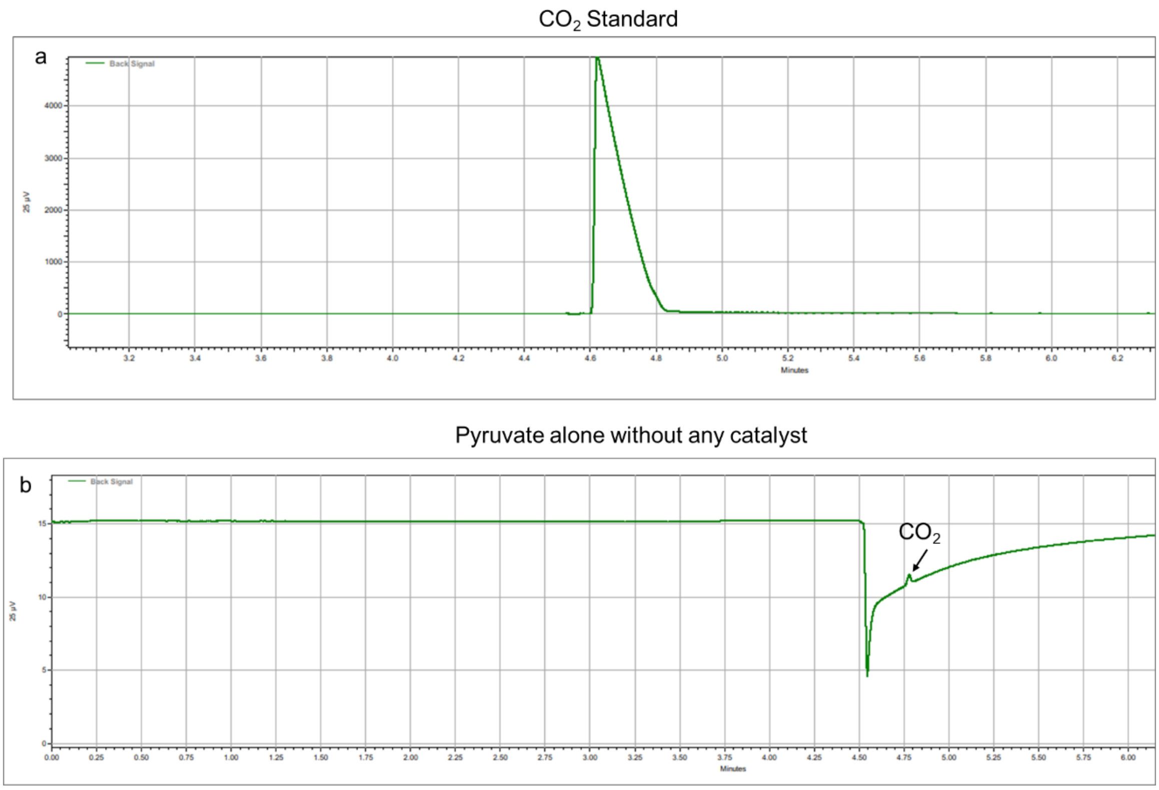

Supplementary Fig. 26. Gas chromatographm of CO_2 standard (a). The CO_2 peak was observed at 4.78 min. Pyruvate (1.0 mg/ml) was shaken for 1 h under 2 bar Ar without any metal catalyst as a control reaction (b). Negligible amount of CO_2 compared to reactions with metal catalysts was observed. For the analysis of CO_2, Thermal Conductivity Detector (TCD) was used.

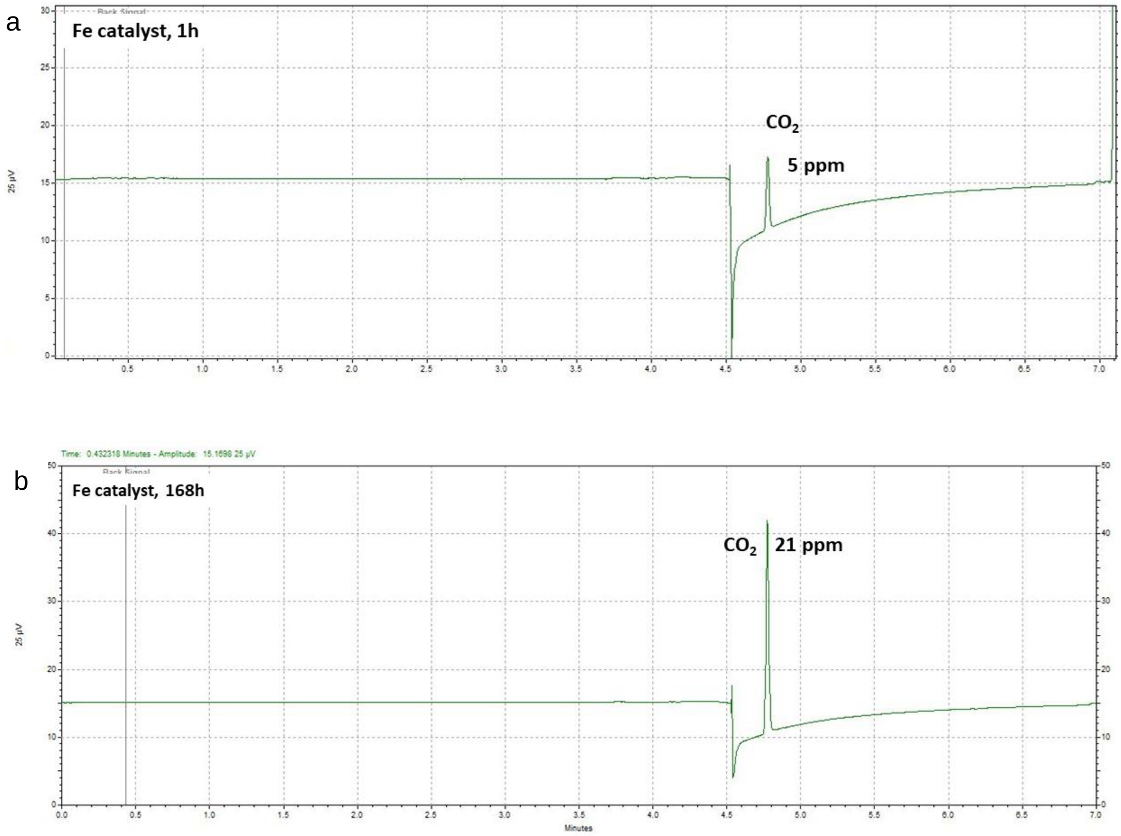

Supplementary Fig. 27. Gas products obtained from pyruvate conversion (1.0 mg/ml) overFe catalyst during 1h (a) and 168h (b) of reaction times at 25 ˚C under 2 bar Ar. The amount of CO_2 after 168h was four times higher than 1h of the reaction time with Fe^0 catalyst.

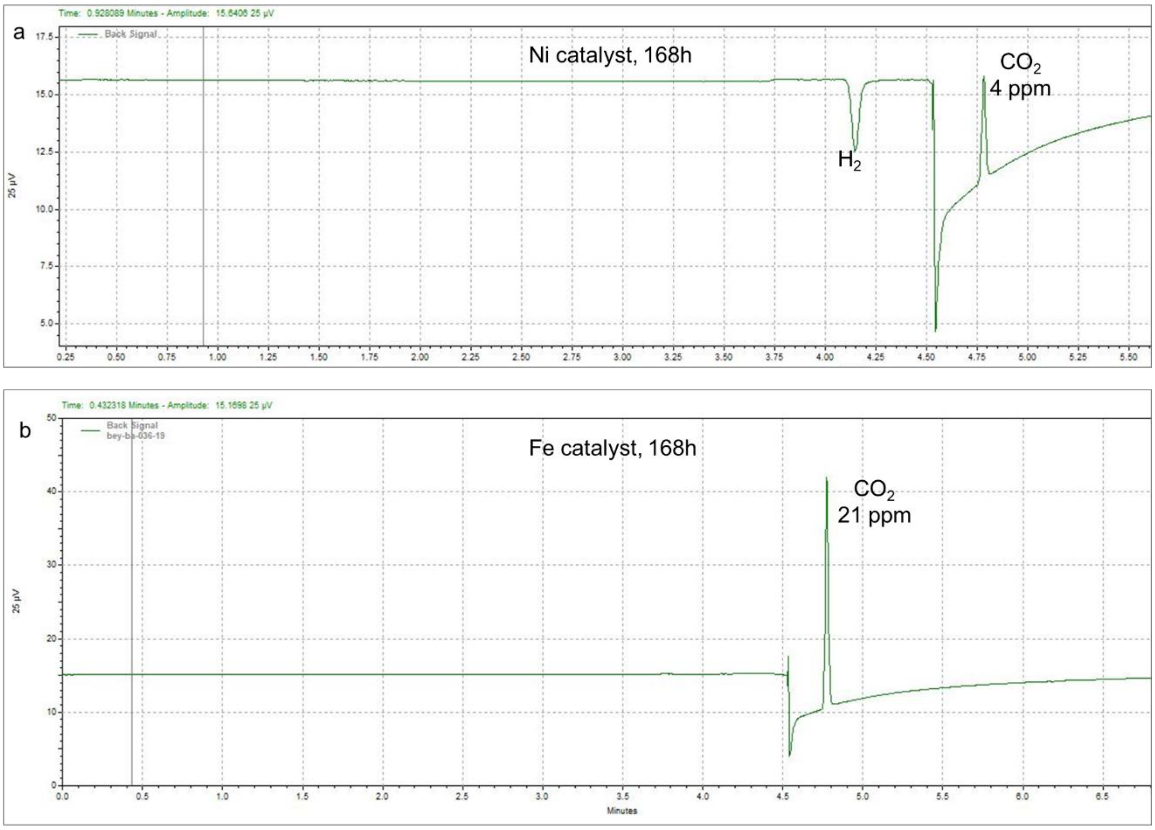

Supplementary Fig. 28. Gas products obtained from pyruvate conversion (1.0 mg/ml) over Ni (a) and Fe catalyst (b) during 168h of reaction times under 2 bar Ar at 25 ˚C. H_2 gas was observed in addition to CO_2 gas with Ni metal catalyst after 168 h. The amount of CO_2 gas after 168 h was higher with Fe^0 catalyst compared to Ni^0.

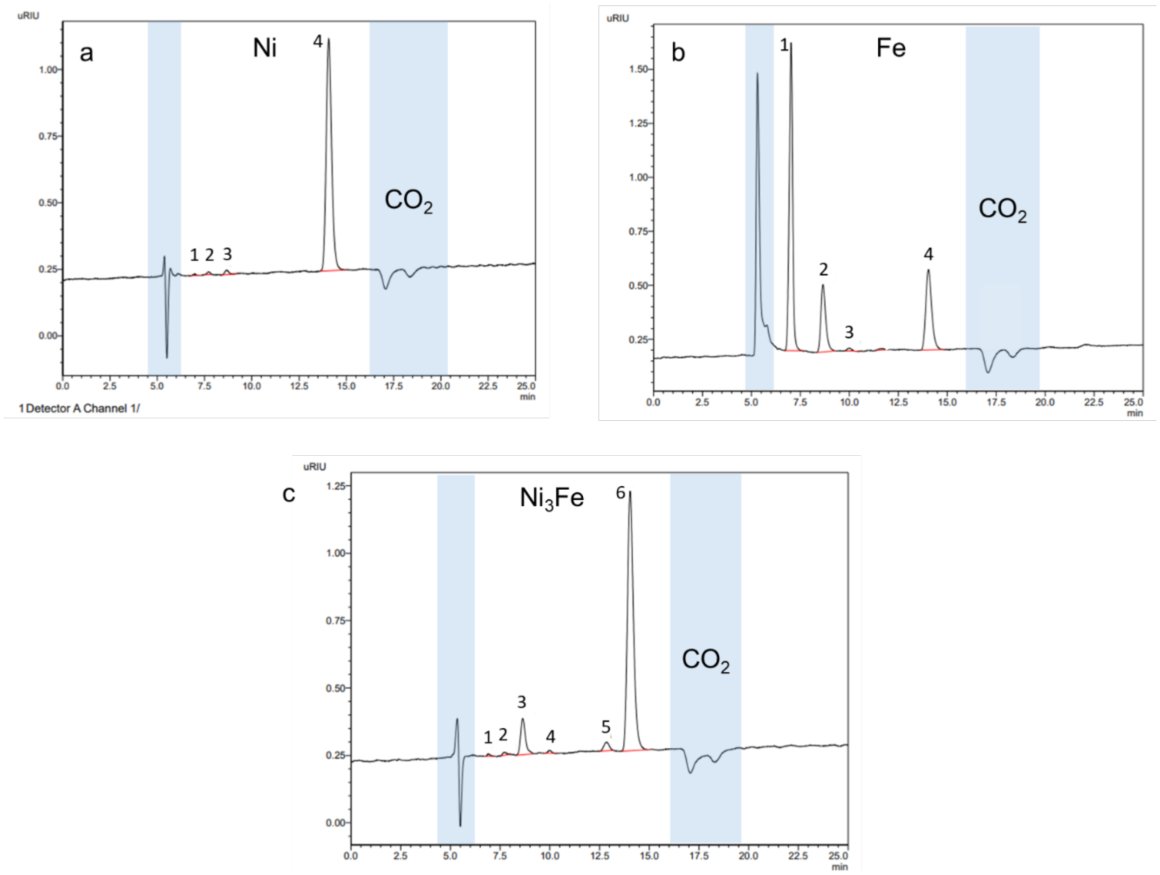

Supplementary Fig. 29. HPLC chromatographs of pyruvate (1.0 mg/ml) conversion over Ni^0 (a), Fe^0 (b), and Ni_3Fe (c) catalysts after 168 h that shows also formation of dissolved CO_2 during the reaction. The amount of dissolved CO_2 after 168 h pyruvate conversion reaction was highest over Fe^0 catalyst and lowest over Ni^0 catalyst. Peaks 1, and 2 in a, b correspond to possible impurities after decomposition and peaks 3 and 4 correspond to citramalate and acetate, respectively. Peaks 1, 2, 3, 5 in c correspond to possible impurities after decomposition, peak 4 and 6 correspond to citramalate and acetate, respectively.

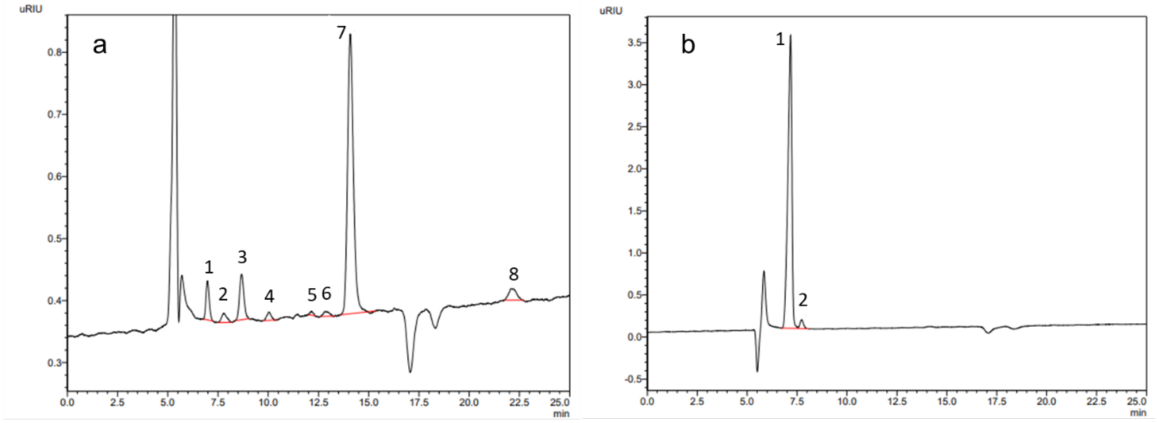

Catalyst	Pyruvate (mg/ml)	Citramalate (mg/ml)	Acetate (mg/ml)	Formate (mg/ml)	Acetone (mg/ml)
Fe NPs	0.01	0.02	0.24	0.00	0.01
Fe bulk	0.76	ND	ND	ND	ND

Supplementary Fig. 30. HPLC results of pyruvate conversion (1.0 mg/ml) after 1h over Fe nanoparticles (a) (peaks 1, 3, 5, 7, and 8 correspond to pyruvate, citramalate, formate, acetate, and acetone, respectively) and Fe bulk catalyst (peak 1 is pyruvate and 2 is water impurity) (b). Higher conversion of pyruvate was observed over nanoparticulate Fe catalyst after 1h. ND: Not detected

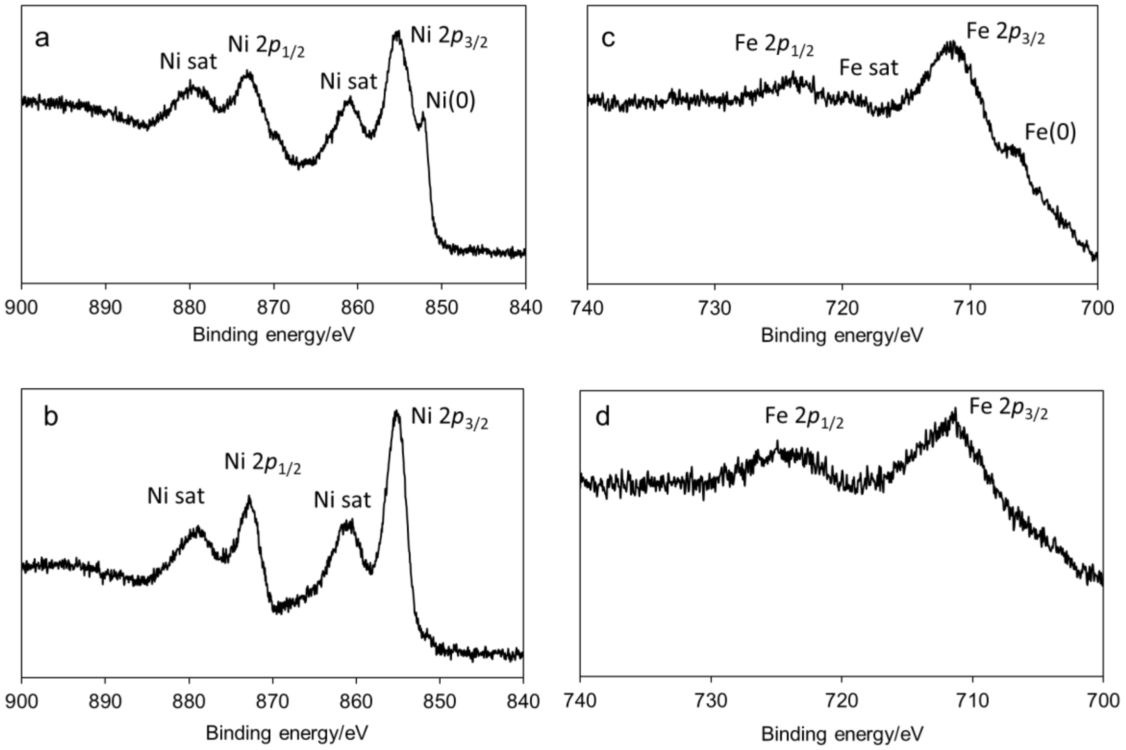

Supplementary Fig. 31. XPS spectra of Ni 2p before the reaction (a), after 168h of the reaction time (b), and Fe 2p before the reaction (c), after the reaction time of 168h (d). After 168h of the reaction time, metallic Fe and Ni were disappeared.

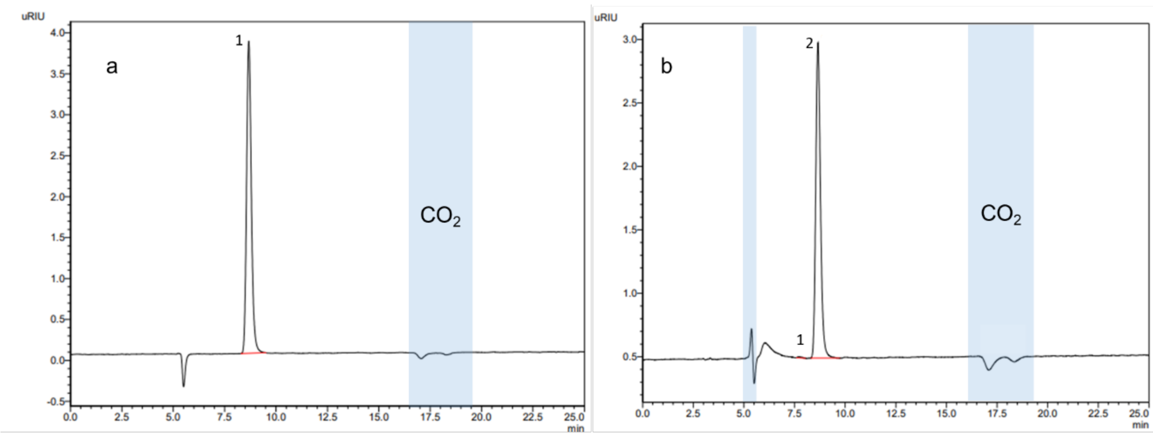

Detector A Channel 1 Peak #	Ret. Time	Area %	Conc.	Units	Name
1	8.68	100.00	0.94	mg/mL	Citramalate
Total		100.00			

Detector A Channel 1 Peak #	Ret. Time	Area %	Conc.	Units	Name
1	7.72	0.32	0.00		
2	8.65	99.68	0.62	mg/mL	Citramalate
Total		100.00			

Supplementary Fig. 32. HPLC chromatographs of citramalate conversion reactions without any metal catalyst (a) and with Ni_3Fe catalyst after 1h (peak 1 is water impurity and 2 is citramalate) (b). Initial concentration of citramalate is 1.0 mg/ml. There was not any conversion without the addition of a metal catalyst.

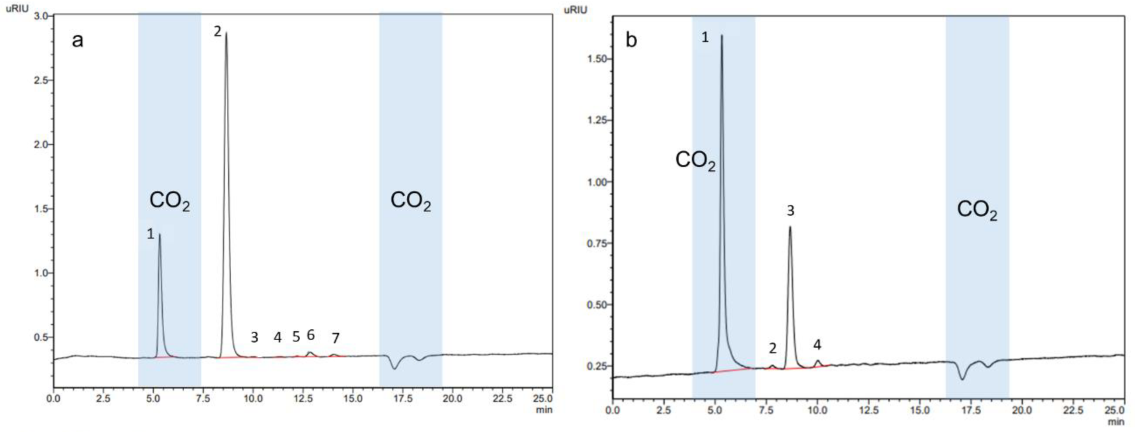

Detector A Channel 1 Peak #	Ret. Time	Area %	Conc.	Units	Name
1	5.33	21.80	0.00		
2	8.66	75.85	0.63	mg/mL	Citramalate
3	10.04	0.17	0.00		
4	11.38	0.14	0.00	mg/mL	Lactate
5	12.20	0.16	0.00	mg/mL	Formate
6	12.87	1.22	0.00		
7	14.05	0.66	0.01	mg/mL	Acetate
Total		100.00			

Detector A Channel 1 Peak #	Ret. Time	Area %	Conc.	Units	Name
1	5.33	65.71	0.00		
2	7.81	0.69	0.00		
3	8.67	32.30	0.14	mg/mL	Citramalate
4	10.02	1.30	0.00		
Total		100.00			

Supplementary Fig. 33. HPLC chromatographs of citramalate conversion over Fe^0 catalyst after 30 min (a) and 1 h (b). After 30 min of the reaction, some intermediate products, such as formate (peak 5), acetate (peak 7), and lactate (peak 4), were observed. Peak 1 corresponds to dissolved CO_2, peak 2 is citramalate in both chromatographs, and remaining low concentration peaks are possible impurities.
